# Splice-switching of the insulin receptor pre-mRNA alleviates tumorigenic hallmarks in rhabdomyosarcoma

**DOI:** 10.1038/s41698-021-00245-5

**Published:** 2022-01-11

**Authors:** Safiya Khurshid, Matias Montes, Daniel F. Comiskey, Brianne Shane, Eleftheria Matsa, Francesca Jung, Chelsea Brown, Hemant Kumar Bid, Ruoning Wang, Peter J. Houghton, Ryan Roberts, Frank Rigo, Dawn Chandler

**Affiliations:** 1 Department of Pediatrics and the Center for RNA Biology, The Ohio State University, Columbus, OH 43210 USA; 2 Center for Childhood Cancer, Abigail Wexner Research Institute, Nationwide Children’s Hospital, Columbus, OH 43205 USA; 3 Resonant Therapeutics, Inc., Ann Arbor, MI 48109 USA; 4 Greenhey Children’s Cancer Research Institute, UT Health, San Antonio, TX 78229 USA; 5 Ionis Pharmaceuticals, Carlsbad, CA 92010 USA

**Keywords:** Targeted therapies, Paediatric cancer

## Abstract

Rhabdomyosarcoma (RMS) is an aggressive pediatric tumor with a poor prognosis for metastasis and recurrent disease. Large-scale sequencing endeavors demonstrate that Rhabdomyosarcomas have a dearth of precisely targetable driver mutations. However, IGF-2 signaling is known to be grossly altered in RMS. The insulin receptor (IR) exists in two alternatively spliced isoforms, IR-A and IR-B. The IGF-2 signaling molecule binds both its innate IGF-1 receptor as well as the insulin receptor variant A (*IR-A*) with high affinity. Mitogenic and proliferative signaling via the canonical IGF-2 pathway is, therefore, augmented by IR-A. This study shows that RMS patients express increased *IR-A* levels compared to control tissues that predominantly express the *IR-B* isoform. We also found that *Hif-1α* is significantly increased in RMS tumors, portraying their hypoxic phenotype. Concordantly, the alternative splicing of *IR* adapts to produce more *IR-A* in response to hypoxic stress. Upon examining the pre-mRNA structure of the gene, we identified a potential hypoxia-responsive element, which is also the binding site for the RNA-binding protein CUG-BP1 (CELF1). We designed Splice Switching Oligonucleotides (SSO) against this binding site to decrease IR-A levels in RMS cell lines and, consequently, rescue the *IR-B* expression levels. SSO treatment resulted in a significant reduction in cell proliferation, migration, and angiogenesis. Our data shows promising insight into how impeding the IGF-2 pathway by reducing *IR-A* expression mitigates tumor growth. It is evident that Rhabdomyosarcomas use *IR* alternative splicing as yet another survival strategy that can be exploited as a therapeutic intervention in conjunction with already established anti-IGF-1 receptor therapies.

## Introduction

Rhabdomyosarcoma (RMS) is the most common soft tissue sarcoma of childhood, with an incidence of 4.5 cases per million children/adolescents per year in the United States^1,2^. It is divided into two main histological variants: embryonal (ERMS, 60–70% of all RMS cases) and alveolar (ARMS, approximately 30% of all RMS cases) RMS. Alveolar rhabdomyosarcoma is characterized by the presence of chromosomal translocation t(1;13)(p36;q14) that results in PAX7–FOXO1 gene fusion; or translocation t(2;13)(q35;q14) that results in PAX3–FOXO1 gene fusion. Approximately, 80–90% of alveolar rhabdomyosarcomas are fusion-positive. Further, the fusion type correlates strongly with the outcome, since *PAX3: FOXO1* is associated clinically with more aggressive tumors than *PAX7: FOXO1*^3^. The remaining two-thirds of RMS (including ERMS and pleomorphic RMS) do not have a pathogenetic translocation and are referred to as fusion negative^4^. Despite improvement in the treatment, there is a substantially poor prognosis for relapse and metastasis patients. Overexpression of IGF-2 (insulin-like growth factor 2) has been found in RMS tumors of both histological subtypes which makes the IGF pathway one of the most frequently deregulated signaling pathways in RMS^5–7^. Targeting the IGF pathway using IGF-1R antibodies has largely failed in phase-III clinical trials owing largely to the lack of understanding of the signaling complexities^8,9^. Therefore, it is imperative to find novel ways to impede the IGF pathway and invent novel therapeutic strategies to treat cancer.

Alternative splicing of the insulin receptor (*IR*), generates two isoforms: the full-length *IR-B* and the exon 11-skipped *IR-A* isoform^10,11^. *IR-B* is highly expressed in adult, insulin-responsive tissues (skeletal muscle, liver, and adipose tissue) and promotes the metabolic effects of insulin^12^. *IR-A*, on the other hand, is predominantly expressed in embryonic tissue and signals proliferative effects^13,14^. The IR is a transmembrane protein from the family of receptors tyrosine kinase which is activated by insulin, IGF-1 and IGF-2; and has an isoform-specific affinity towards each ligand. *IR-A* and *IR-B* have similar affinities for native insulin but differ substantially in their affinities for IGF-1 and IGF-2^10^. The increased affinity of *IR-A* toward IGF-2 orchestrates a cascade of signals involved in numerous developmental and mitogenic pathways^15^. Increased expression of IGF-2 and the consequent over-activation of this pathway by both insulin and IGF-2 is highly prevalent in cancer cells and may represent an important factor of resistance to various anti-cancer drugs^10,16,17^. This is particularly important in RMS because both fusion-positive and fusion negative tumors have been shown to have an increased expression of IGF-2 resulting either by loss of imprinting or loss of heterozygosity in the 11p15.5 chromosomal region^18,19^. These genetic malfunctions make it imperative to discover novel treatments to block this pathway in RMS. Treatment resistance is linked to the ability of IGF-2 to circumvent IGF-1R by signaling through an alternatively spliced form of *IR-A*. The urgent need is that of a combinatorial treatment wherein we could block the IGF-1R on one hand and also revert the splicing of *IR-A* back to the full-length *IR-B* so as to completely block the downstream mitogenic signaling of IGF-2 and suppress the cancer phenotype.

A study performed using an IR minigene, consisting of exons 10–12, reported SRSF3 (SRp20) and CUG-BP1 (CELF1) as potential negative and positive splicing regulators respectively, mediating IR alternative splicing^20^. However, not much is known about the interplay of these RNA-binding proteins and their response to malignant stress. In this work, we show that pediatric RMS patient samples, as well as RMS cell lines, demonstrate an increased expression of *IR-A* compared to normal muscle tissue. Concordantly, IR isoform A expression increases in hypoxic conditions consistent with earlier reports that hypoxia decreases insulin signaling^21^. Furthermore, we show that splice-switching oligonucleotides (SSOs) are able to reverse this splicing change. This decrease in the expression of the *IR-A* isoform results in significantly reduced proliferation, migration, and angiogenesis of RMS cell lines. Overall, our data delineate a novel strategy to mitigate tumor signaling by modulating the splicing of IR from *IR-A* to *IR-B*.

## Results

### The alternately spliced insulin receptor A isoform is prevalent in RMS patient samples and cell lines

**Fig. 1:**
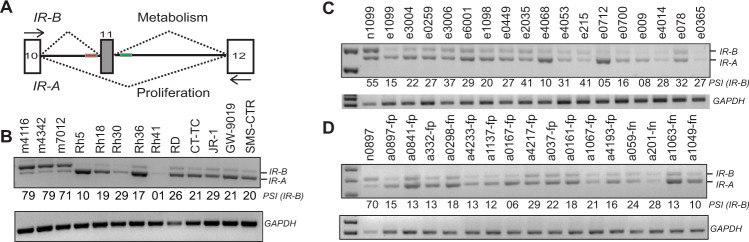
RMS cell lines and patient samples express increased levels of *IR-A*. **A** Schematic depicting the splicing of insulin receptor gene. **B** RT-PCR of control muscle samples and RMS cell lines depicting the two insulin receptor (IR) isoforms *IR-A* and *IR-B*. Cells were cultured in the required medium, RNA was extracted, and PCR was performed using the primers depicted by black arrows in (**A**). The PCR products were run on a 2% agarose gel and bands were quantified. RT-PCR was performed on (**C**). Embryonal (e) and **D** Alveolar (a) RMS samples (depicted with different numbers) using the primers for IR depicted in (**A**) (black arrows). Samples from normal adjacent tissue (for one sample) are shown as control (*n*). The fusion status (fp = fusion positive, fn = fusion negative) status is denoted on the alveolar tumor samples. All embryonal samples were also tested and found fusion negative. The percentages for *IR-B* are shown as PSI (percent spliced in). GAPDH is shown as a loading control. Results are shown as the standard error of the mean (±SEM).

The *IR* gene is composed of 22 exons. Whereas all exons are included in most normal adult tissues, exon 11 is excluded during embryogenesis^22^. Exon 11 of the *IR* gene is composed of 36 nucleotides that encode for 12 amino acids residing at the C-terminal of the receptor alpha subunit. Though only a small change in the protein composition, deletion of these 12 amino acids results in a receptor that has increased binding affinity for IGF-2 and is capable of responding to autocrine and paracrine signaling^11^ (Fig. 1A). The expression of these isoforms is regulated during development and is altered in some breast and liver cancers^6,23,24^. RMS is characterized by high levels of IGF-2, produced in an autocrine manner^15,25,26^. It is evident that IGF-1R and its ligands play roles in the proliferation and survival of tumor cells. Although there have been efforts to target and block the IGF-2 pathway by either using IGF-1R or AKT/mTOR inhibitors, only low response rates have been achieved^9,26^. This can be attributed to multiple reasons, but primarily because single-agent therapies are insufficient, short-lived, and some of them display high toxicity. Additionally, this lack of efficacy could result from compensatory signaling of *IR-A* binding to IGF-2^27^. Based on these observations, we hypothesized that RMS tumors would display aberrant alternative splicing of the IR pre-mRNA and sought to understand its splicing pattern in these specific tumors. To this end, we analyzed normal muscle samples and multiple RMS cell lines and performed a reverse transcription-polymerase chain reaction (RT-PCR) for the *IR* gene. The primers flanking exon 11 amplify two products: full-length *IR-B* and smaller exon 11-lacking isoform *IR-A*. By calculating ratios of these products, we found that the expression of *IR-B* is decreased from an average of 76.3% (±SEM 2.66) in normal muscle to 19.3% (±SEM 2.72) in RMS cell lines. We further performed this analysis in control muscle tissue and a cohort of 30 rhabdomyosarcoma patient samples comprising both the fusion-positive and fusion negative subclassification^28^. In 100% of the tumor samples, we witnessed an increased expression of the *IR-A* isoform compared to the normal controls (Fig. 1B–D). The percent increase in *IR-B* has been quantitated and represented as PSI (percent spliced in). These data show that RMS tumors change the splicing pattern of IR to decrease PSI resulting in more *IR-A* with exon 11 excluded.

### Hypoxia increases the expression of *IR-A* isoform and mutation of the hypoxia response element in the minigene construct prevents it

**Fig. 2:**
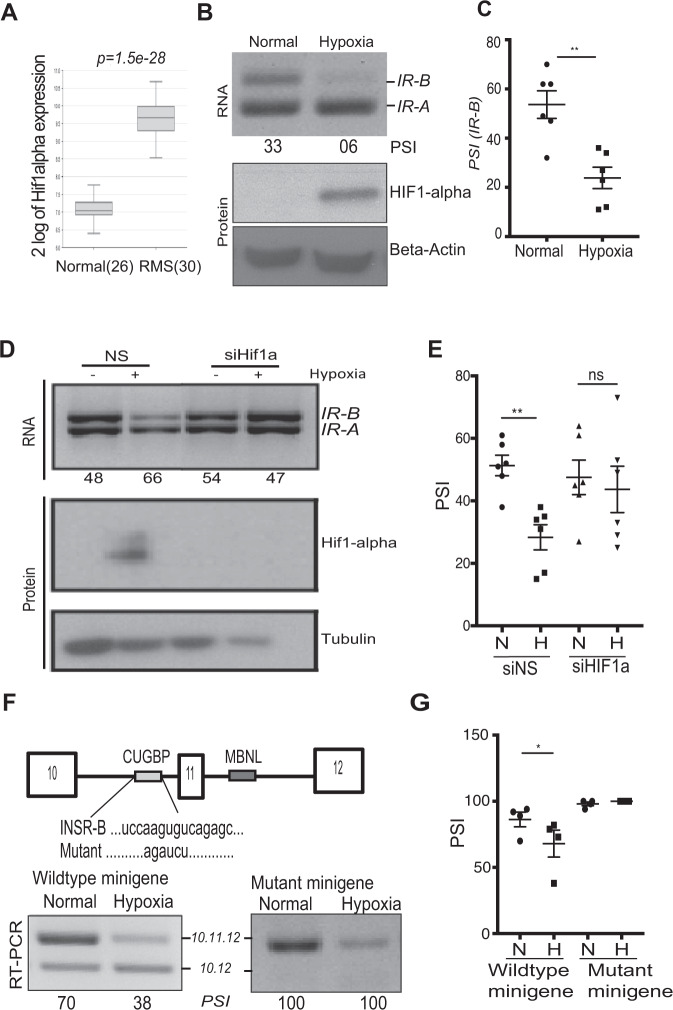
Hypoxic stress increases the expression of *IR-A* isoform. **A** The Gene expression data for HIF-1α expression were extracted from *Schafer-Welle* (*Scafer-Welle-56-MAS5.0-u133a*) dataset using R2: Genomics Analysis and Visualization platform for 26 normal muscle samples and 30 RMS samples^35^. The data distribution is represented by a box plot, the line inside the box represents the median and the whiskers represent minimum and maximum expression. *p* = 1.5e−28. **B** RT-PCR depicting the IR alternative splicing in HeLa S3 cells after 48 h of normoxia or hypoxia (1% O_2_). Western blot analysis of the Hif-1α induction by hypoxia treatment is shown with β-actin as control. **C** Quantification of *IR-B* expression in control and hypoxic conditions; *n* = 6, *p* = 0.0018. **D** RT-PCR analysis for the INSR alternative splicing of the siRNA-mediated knockdown of Hif-1α followed by treatment of normoxia or hypoxia (1% O_2_). Western blot analysis for Hif-1α expression and β-tubulin as a loading control. **E** Quantification of *IR-B* expression in control and hypoxic conditions of the Hif-1α knockdown experiments. *n* = 6, *p* = 0.0013. **F** Depiction of the wild-type insulin receptor (*IR*) minigene or the CUG-BP1 binding-site mutated minigene. RT-PCR depicting both INSR minigenes alternative splicing. **G** Quantification of the 10.11.12 isoform expression is shown *n* = 4, *p* = 0.405. Results are shown as the standard error of the mean (±SEM).

Solid tumors are characterized by both acute and chronic hypoxia, making the ability of tumor cells to adapt to hypoxia essential for tumor progression^29^. In addition to disruption in IGF signaling pathways, hypoxia is a well-known feature of aggressive pediatric tumors^30–32^ including RMS^33,34^ and correlates significantly with the patient outcome^35^. Hypoxia alters alternative splicing and hypoxia-induced alternative splicing has been also called the 11th hallmark of tumorigenesis^36–38^. Moreover, hypoxia is known to interfere with insulin signaling in various cell types^21,39^. To better understand the relevance of the tumor hypoxia in RMS, we interrogated the Schafer-Welle 56-MAS5.0-u133a data set in which 26 normal muscle samples were compared with 30 RMS patient samples (15 ARMS, 15 ERMS) and found that RMS patient samples significantly overexpress *HIF-1α*, a characteristic feature of hypoxia (Fig. 2A)^40^. In order to directly assess the changes in *IR* splicing in response to hypoxia, we grew cells in either hypoxic (1% O_2_) or normal conditions (21% O_2_) and harvested RNA to quantify the *IR-A*/*IR-B* ratios. Because RMS cells already express predominantly the *IR-A* isoform, we turned to the standard laboratory cell line *HeLa S3* as it exhibits a relatively equal expression of the two IR isoforms. An equal number of cells were seeded and maintained in either hypoxia (1% oxygen, 24–48 h) or normoxia conditions. After 48 h, cells that underwent hypoxia treatment had significantly increased *IR-A* induction as compared to the control cells (Fig. 2B, C). To assess hypoxia induction, we blotted for *HIF-1α* and found it to be upregulated in hypoxia-treated cells (Fig. 2B). In order to determine whether *HIF-1α* expression is necessary for this splicing event, we knocked down *HIF-1α* using siRNA. We found that on knocking down *HIF-1α* in HeLa cells the hypoxia-mediated *IR* splicing shift towards *IR-A* is abrogated showing that *HIF-1α* is important for mediating this splicing event (Fig. 2D, E). Our results indicate that hypoxic stress induces the expression of the *IR-A* isoform in cancer cells. Our findings support the idea that RMS cells induce differential splicing of the *IR* gene in order to adapt to the hypoxic microenvironment and potentially promote tumorigenesis.

In order to better understand the hypoxia-induced regulation of the *IR* alternative splicing process, we obtained an IR minigene, which is comprised of exons 10–12^20^. We transfected RMS cells with the *IR* minigene and subjected them to hypoxic conditions. Once again, exclusion of exon 11 is increased after hypoxia treatment showing that the minigene retains the hypoxia-responsive alternative splicing, similarly to the endogenous gene. To interrogate the role of CUG-BP1 (CELF1), a known regulator of the *IR* splicing event^20^, we used a minigene with a deletion of the CUG-BP1 binding site and found that the *10.12* isoform could no longer be induced, as compared to the wild-type construct (Fig. 2F, G). Hence the CUG-BP1 binding site in the IR intron 10 contains a potential hypoxia-responsive element, which is essential for the hypoxia-induced splicing event.

### Splice switching oligonucleotide (SSO) treatment restores *IR* splicing to *IR-B*

In order to determine whether we could exploit this splicing process as a therapeutic vulnerability and target the IGF-2 pathway in rhabdomyosarcoma, we employed SSO technology. Based on the information of hypoxia-responsive element (Fig. 2), as well as the CUG-BP1 binding sites in the IR pre-mRNA^20^ we designed SSOs to include or exclude exon 11. SSOs are short, synthetic, antisense, and modified nucleic acids that base pair with the pre-mRNA sequences at the splicing factor binding sites and alter splicing. SSOs have been used successfully in the past both to decrease exon inclusion (in the muscular dystrophy-causing gene *DMD*) and to increase exon inclusion (exon 7 of the spinal muscular atrophy [SMA] causing gene *SMN*) and are now in clinical trials and also approved by FDA for SMA therapy^41^. We collaborated with *Ionis Pharmaceuticals* for SSO design and performed an SSO walk spanning the regions in and around exon 11. We designed SSOs to increase the inclusion of exon 11 by masking the negative regulatory element CUG-BP1 binding site within the *IR* pre-mRNA. We also designed SSOs to exclude exon 11 by masking a downstream positive regulatory element MBNL1 binding site within the intron 11 of the *IR* pre-mRNA^20^. We co-transfected HeLa S3 cells with an *IR* minigene as well as with the designated SSO. Since the therapeutic application of the antisense oligos requires exon 11 to be included, we started off by screening the region flanking the CUG-BP1 binding site in intron 10 and within exon 11 itself.

**Fig. 3:**
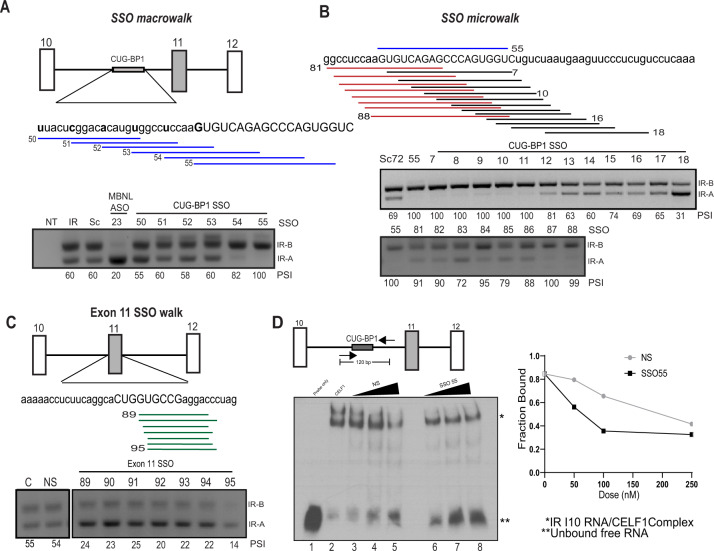
Splice switching oligonucleotide (SSO) treatment restores *IR* splicing to *IR-B*. **A**, **B** Representation of the insulin receptor minigene. The CUG-BP1 binding site is shown on the *IR* pre-mRNA. The blue lines represent the SSOs used for the macro-walk with the new SSO starting at the “bold” letter in the sequence. The red and black lines represent the SSOs used for the micro-walk. RT-PCRs depicting the IR isoforms in the presence of control SSOs or SSOs that target either the CUG-BP1 binding site are shown below the sequences. The capital letters in the sequence represent the binding site for SSO55. The percent exon 11 inclusion is shown as PSI, percent spliced in. **C** Representation of exon 11 SSOs (green lines). RT-PCR using SSOs on the second CUG-BP1 binding site in exon 11 is shown below. **D** Schematic to represent the region amplified and used as a probe for the RNA electrophoretic shift assay from the IR intron 10. The complex formed by CUG-BP1 and the I10 probe is depicted as * and the unbound I10 probe is depicted as **. Experiments were performed in triplicate and quantitated in the graph on the right. The bound fraction was calculated as (bound complex intensity)/(bound complex intensity + unbound probe intensity). Results are shown as the standard error of the mean (±SEM).

First, we performed a “macro-walk” using six overlapping SSOs (SSO50–SSO55), represented by blue lines. This macro-walk spans the entire CUG-BP1 binding site region in 5-nt increments and targets a 40–50 nucleotide region. Out of the six SSOs, the last two were capable of inducing *IR-B* expression from 60 to 82%, with SSO55 reaching the maximum effect and increasing the *IR-B* expression to 100% (Fig. 3A). In order to pinpoint the most effective SSO targeting this sequence element and allowing the robust expression of the desired isoform, we designed an “SSO micro-walk” around the region of SSO55 by using 20 additional consecutive SSOs, represented by red and black lines (SSO micro-walk) at a 2-nt resolution to cover the entire 40-nucleotide region. Based on the percent expression of *IR-B* in these cells, we were able to determine that SSOs (55, 87, and 88) were highly effective in mediating this splicing change to restore the expression of *IR-B* (Fig. 3B). We also performed a consecutive SSO walk to block the 30-nucleotide element in exon 11 but were unable to target this second CUG-BP1 binding site. Our inability to target the element within exon 11 was most likely due to the small exon size and interference with the U1 and U2 snRNPs that recognize the splice sites flanking the exon or the interaction with other splice regulatory elements within the exon (Fig. 3C). Additionally, in order to test whether we can exclude exon 11 by blocking the MBNL1 binding site, a positive regulator of *IR* splicing, we performed an SSO walk on intron 11. We used 20 SSO sequences across the MBNL1 binding site and found that SSO23 performed the best causing up to 95% exclusion of exon 11 (Supplementary Fig. 1A–C and Fig. 3A). From our SSO walk, we determined that SSO55 outperformed the other SSOs with respect to IR-B production and moved forward to further characterize its functionality.

In order to determine the specificity of splice SSOs, we used the program Bowtie^42^ to predict off-targets for the SSO55 sequence in the human transcriptome. The existing literature for oligomer design and chemistry optimization recommends at least 16-nucleotide oligomers for optimal binding^43,44^. Therefore, using *E*-value and percent identity as parameters, we found that the SSO55 sequence does not bind to any DNA sequences in the genome except the *INSR* genomic region with 100% complementarity (18/18 nt). We were, however, able to identify additional binding sites in the human genome with one or two mismatches (Supplementary Table 2). Gene downregulation events are not anticipated given that SSOs that modulate splicing do not induce RNase H- or AGO2-mediated mRNA degradation.

In order to determine whether the SSO55 disrupts the binding of CUG-BP1 to intron 11^20^, we performed an electrophoretic mobility shift assay (EMSA). Briefly, we used in vitro transcribed RNA probes containing the CUG-BP1 binding site in the *IR* intron 10, with recombinant CUG-BP1 protein, to form IR pre-mRNA: CUG-BP1 complex. Our data show that CUG-BP1 forms a complex with a specific region of IR intron 10. Furthermore, increasing concentrations of SSO55 (compared to NS) more efficiently decreases the amount of the bound CUG-BP1: *IR* intron 10 complex, evidenced by decreased levels of the slower migrating band in the presence of low concentrations of oligonucleotide in lanes 6 and 7 compared to lanes 3 and 4 (Fig. 3D, E). SSO55 which binds the CUG-BP1 binding site causes the destabilization rapidly at much lower concentrations as compared to the control NS SSO (shown in the right panel of 3D), showing that CUG-BP1 is an important protein that mediates the splicing changes in *IR* pre-mRNA and that the SSO disrupts its binding, changing the splicing outcome to allow for the expression of *IR-B*.

Importantly, in order to assess if SSO55 was potentially binding to other CUG-BP1 targets, we used the published Xia et al.^45^ RIP-seq datasets that identified global targets of CUG-BP1 by determining the RBP binding sites in HeLa cells. We tested several of these genes (*LMO7*, *PARD3*, and *ZDHHC16*) and performed RT-PCR to look for changes in alternative splicing in the presence of SSO55. We, however, did not witness any changes, showing that the effects we see with SSO55 are specific to the splicing change of IR (Supplementary Fig. 4). We also cross-referenced the identified CUG-BP1 binding sites with binding sites containing 1–2 mismatches identified above by Bowtie. Although we found 17 genes with both an SSO mismatch and CUGBP binding site, none of the binding sites are located in a position to be reasonably expected to control a nearby regulated exon.

Given our data that SSO55 can effectively and specifically block CUG-BP1 from binding to the hypoxia response element in the *IR* pre-mRNA and modulate the alternative splicing pattern of *IR*, and considering the relevance of the IR signaling pathway in cancer, it is important to check whether these splicing changes are also functionally pertinent.

### SSO treatment shifts splicing back to *IR-B* and helps decrease proliferation and migration in RMS cell lines

**Fig. 4:**
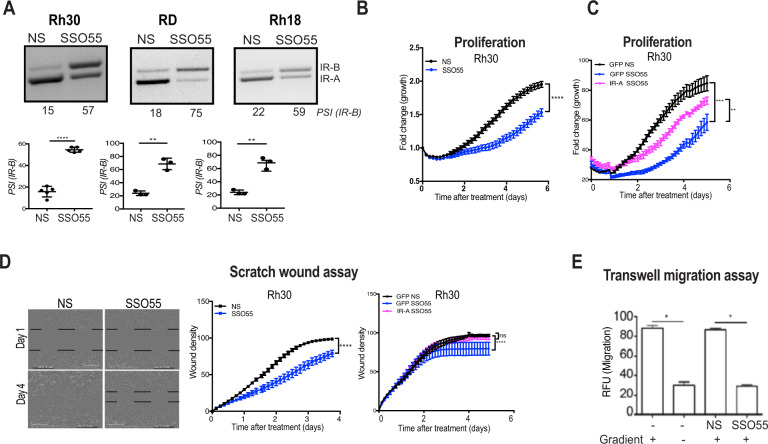
SSO treatment decreases proliferation and migration in RMS cell lines. **A** Non-specific (NS) and SSO55 were transfected endogenously into Rh30, RD, and Rh18 cells, and RT-PCR for *IR* was performed after 24 h. The percent expression of IR-B is shown as PSI. The graph below the gel picture shows the quantification from *n* = 5, *n* = 3, and *n* = 3 experiments, respectively. *p*-Value Rh30 < 0.001, RD = 0.0002, Rh18 = 0.0012. **B** Proliferation: Rh30 cells were seeded, transfected with either NS or SSO55, and subjected to proliferation assay using the Incucyte^®^ software; *p*-value**** < 0.0001 using 2-way ANOVA. **C** Rh30 cells were seeded, transfected with either *IR-A-GFP* or control *GFP* and NS SSO or SSO55 and subjected to proliferation assay using the Incucyte^®^ software; *p*-value*** = 0.0005 and ** = 0.0059 using 2-way ANOVA. **D** Wound healing assay: Rh30 cells were seeded in a 96-well plate and transfected with either non-specific (NS) or SSO55. After 24 h, the Incucyte^®^ wound maker was used to make the wound and the migration of cells was measured using the Incucyte^®^ platform. The wound density numbers are quantified, *p*-value**** < 0.0001 using 2-way ANOVA. Rh30 cells were seeded in a 96-well plate and, transfected with either IR-A-GFP or GFP and NS SSO or SSO55. After 24 h, the Incucyte^®^ wound maker was used to make the wound and the migration of cells was measured using the Incucyte^®^ platform. The wound density numbers are quantified. Statistics are calculated using 2-way ANOVA, *p*-value**** < 0.0001. **E** Trans-well migration assay: Rh30 cells were transfected with NS or SSO55 and placed in the top chamber of a dual-chamber Boyden assay and exposed to an FBS gradient or not. Non-transfected Rh30 cells were used as control. Percent migration was quantified. *n* = 4 for each group, an asterisk indicates *p* = 0.05 in one-way ANOVA with Holm–Sidak post hoc analysis. Results are shown as the standard error of the mean (±SEM).

As shown in Fig. 1, RMS tumors and cell lines predominantly express *IR-A* hence we have used the RMS cell lines Rh30, RD, and Rh18 to query the ability of SSO to alter splicing in RMS cells. While minigene constructs are highly useful tools for identification and in-vivo analysis of regulatory elements for splicing control, it is imperative to understand the splicing processes in the context of endogenous full-length pre-mRNA due to possible disparities related to the length and the structure of the minigene. In order to determine whether the SSO sequences are able to regulate splicing in the context of the endogenous full-length pre-mRNA, we transfected the SSO55 endogenously in RMS cells and performed a dose-response assay ranging from 0 to 250 nM of SSO. RT-PCR for the *IR* gene of these cells depicted a 0 to 60% increase in *IR-B* isoform and therefore restored the splicing in these cells in an endogenous setting (Supplementary Fig. 2A–C). Furthermore, based on SSO efficacy as well as cell viability, we used 100 nM as the optimal dose for future experiments. We transfected the SSO into three RMS cell lines and observed a robust and significant decrease in *IR-A* expression. The RT-PCR for the *IR* gene showed that SSO55 successfully increased the *IR-B* isoform from 15 (±SEM 2.1) to 54% (±SEM 1.0) in Rh30; 18 (±SEM 3.4) to 74% (±SEM 2.9) in RD and 22 (±SEM 2) to 64% (±SEM 5.0) in Rh18 cells in an endogenous setting (Fig. 4A).

Because the transfection of SSO55 into RMS cell lines effectively promoted exon 11 inclusion and decreased *IR-A* splice isoform levels, we next assessed whether the restoration of this splicing helps alleviate cancer cell hallmarks in RMS cells. Since the molecular weight of IR-B and IR-A are 130 and 129 kDa, respectively, and are indistinguishable on western blot, we assessed the presence of the proteins in functional assays. We performed proliferation assays on RMS cell lines that were transfected with either a control NS SSO or SSO55, specific for *IR-A* reduction. We found that there was a significant decrease in proliferation of Rh30 cells transfected with SSO55 as compared to the control cells (*p* < 0.0001) (Fig. 4B). Similarly, other RMS cell lines RD and Rh18 showed a significant decrease in proliferation (*p* < 0.01 and *p* < 0.001), respectively (Supplementary Fig. 3). In order to determine whether the effect we see in proliferation is specifically because of SSO55 reversing the splicing pattern of *IR*, we transfected the cells with a control *GFP* plasmid and splicing resistant *GFP-IR-A* plasmid followed by SSO transfection and performed our proliferation assay. Our data show that in the presence of splicing resistant *IR-A* plasmid, the decrease in proliferation of Rh30 by SSO55 is effectively rescued showing that the functional change in growth in the cells is, at least in part, a consequence of the change in IR splicing (Fig. 4C, Supplementary Fig. 5A, B). To determine whether the splicing shift toward the *IR-B* isoform also inhibits cell migration, another cancer hallmark, we performed scratch-wound assays in Rh30 cells transfected with either SSO55 or NS SSO. We found that while the control cells migrated faster and closed the gap in 90 h, the cells transfected with SSO55 showed a decreased migration rate and failed to close the gap within that time (Fig. 4D). We repeated the scratch-wound migration assay using cells that were transfected with the splice-resistant *GFP-IR-A* plasmid and found that the migration rates of the cells transfected with SSO55 were rescued by the concomitant expression of the IR-A plasmid (Fig. 4D). Additionally, we performed quantitative trans-well migration assays and found that SSO55 decreased the migration of Rh30 cells sixfold (Fig. 4E). As seen for proliferation and scratch wound assays above, the overexpression of *IR-A* reinstates the migrating ability of the cells in the presence of SSO55 (Supplementary Fig. 3B).

Our data demonstrate that shifting the splicing of the IR back to *IR-B* isoform inhibits proliferation and migration of RMS cells, two important attributes of cancer.

### SSO treatment diminishes tube formation and angiogenesis in RMS cells

**Fig. 5:**
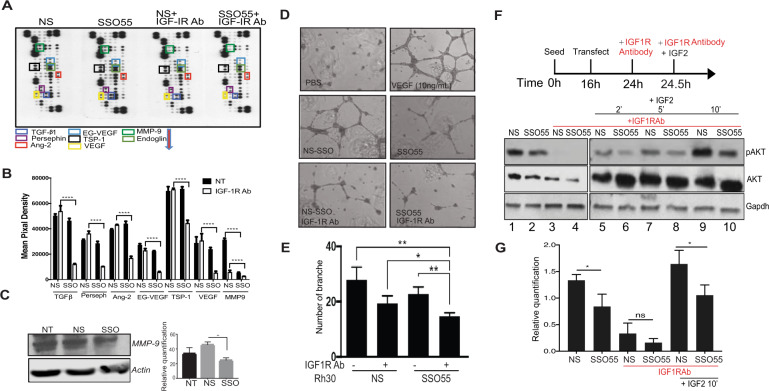
SSO treatment diminishes tube formation and angiogenesis in RMS cells. **A** A protein profiler array was used to assess the expression of angiogenic factors, and the expression was quantified. **B** Quantification for *n* = 2, statistics were done using 2-way ANOVA ****p < 0.0001. **C** Rh30 cells were treated with NS and SSO55 compounds and lysed for Western blot analysis. MMP-9 and Actin are shown. Quantification of MMP-9 expression, *n* = 3, Mann–Whitney test. **D** Human umbilical vein endothelial cells (HUVEC) were incubated with either PBS or media from Rh30 cells treated with either NS or SSO55 alone or in combination with IGF-1R antibody. **E** The vessels/branch formation was measured and quantified using CD31 antibody staining. **F** Rh30 cells were seeded in a six-well plate, transfected with NS or SSO55. After 24 h, all wells but the controls were treated with IGF-1R antibody (depicted in red). After 30 min, one NS and SSO55 treated well was harvested and IGF-2 was added to the remaining wells. The wells were harvested with RIPA buffer supplemented with protease and phosphatase inhibitors after 2, 5, and 10 min. The blots for p-AKT (MW ~60 kDa), total AKT (MW ~60 kDa), and GAPDH (MW ~37 kDa) are depicted. The relative quantification (pAKT/ loading control) was done using ImageJ and is shown in the graph (**G**). Statistics for *n* = 3, paired *t*-test, *p*-value = 0.357 and 0.417, respectively. Results are shown as the standard error of the mean (±SEM).

Rhabdomyosarcoma is characterized by local invasion and metastasis and RMS cell lines are known to secrete angiogenic factors like VEGF and bFGF^46,47^. Additionally, increased IGF-2 levels in this tumor type contribute to increased angiogenesis^15^. Related studies from the Pediatric Preclinical Testing Program found that the IGF-1R inhibitor shows anti-angiogenic effects^48,49^. However IGF-1R-blocking monoclonal antibodies showed poor response rates in the late phase clinical trials^8,26^. In order to check whether SSO55 can affect the expression of certain angiogenesis modulators in RMS cells, we used an R&D systems proteome profiler array that features a nitrocellulose membrane with immobilized biotinylated antibodies. We incubated the membrane with lysates from Rh30 cells that were transfected with non-specific SSO or SSO55 in the presence or absence of an IGF-1R antibody. The proteins were detected using the streptavidin–horseradish peroxidase (HRP) detection method (Fig. 5A, B). Our data show that MMP-9, a matrix metallopeptidase previously shown to be involved in tumor vascularization and metastasis^50,51^ is decreased eightfold in the presence of SSO55 and reduced to near undetectable levels in the presence of both SSO55 and IGF-1R-blocking antibody. The decrease in MMP-9 levels is confirmed by Western blot (Fig. 5C). MMP-9 has been shown to positively regulate the IR survival pathway^52^ and therefore its abrogation with the SSO works favorably to inhibit the growth of cancer cells. No other protein on the proteome array showed changes on either treatment with IR antibody or SSO alone. However, another 6 out of 55 proteins represented showed a significant decrease in the presence of both SSO55 and IGF-1R antibody and these included the major angiogenic modulators such as VEGF, TSP-1, TGFb1, all known to act downstream of IGF-1R (Fig. 5A, B).

Angiogenesis is characterized by a number of cellular events including endothelial cell migration, invasion, and differentiation into capillaries. In vitro endothelial tube formation assays are used as a model for studying endothelial differentiation. In order to determine whether this decrease in the angiogenic factors (Fig. 5A, B) affects the tube formation in endothelial cells, we established 2D co-cultures of human umbilical vein endothelial cells (HUVEC) with Rh30 and RD cells. Briefly, HUVECs were plated with extracellular matrix and conditioned media from Rh30 or RD cells treated with either NS or SSO55 with or without an IGF-1R antibody. The formation of capillary structures called tubes was measured using an inverted microscope. Our data show that the treatment of IGF-1R antibody alone or the SSO55 alone did not inhibit the endothelial cells to form capillary structures; however, the number of branches was significantly decreased in cells treated with a combination of SSO55 and the IGF-1R blocking antibody (Fig. 5D, E, Supplementary Fig. 6A, B). In order to discern whether the effect on endothelial cells branching is specific to the effect of SSO55 on *IR* splicing, we repeated these experiments in the presence of splice-resistant *GFP*-*IR-A* plasmid. The data shows that the *GFP-IR-A* plasmid restores the capillary forming ability of HUVECs showing that SSO55 is specific in mediating this effect on branch formation by changing the IR splicing (Supplementary Fig. 6C, D).

These results suggest that while none of the individual therapies are sufficient to inhibit angiogenic protein expression and tube formation, the combination treatment comprising of the IGF-1R blocking antibody plus SSO55 could prove to be highly advantageous to block the angiogenic signaling.

The IR binds to insulin and/or IGF-2 resulting in a cascade of phosphorylation events which results in the phosphorylation of AKT and activation of mitogenic pathways with major roles in cell survival and growth. The hyperactivation/phosphorylation of AKT has been shown to be a characteristic feature of many cancers, most often leading to aggressive tumors^53–55^. We thus tested whether the addition of SSO, with or without the IGF-1R antibody can inhibit this pathway by inhibiting the phosphorylation of AKT. In order to optimize the conditions for this experiment, we performed a pilot experiment employing multiple different time-points and treatments using both IGF-1 and IGF-2 which allowed us to design a roadmap for future experiments (Supplementary Fig. 7). Our data from the pilot experiment showed that serum starvation attenuates AKT phosphorylation (Supplementary Fig. 7A lanes 1 and 2). IGF-2 activates p-AKT signaling more than IGF-1 (Supplementary Fig. 7A lanes 3 and 4) and that in serum-starved conditions p-AKT signaling does not change in response to NS SSO or SSO55 addition (Supplementary Fig. 7A lanes 6 and 7). We also found that in the presence of IGF-2 and not IGF-1, SSO55 decreases the p-AKT levels either alone (Supplementary Fig. 7A lanes 8–11) or in combination with the IGF-1R Ab (Supplementary Fig. 7A lanes 12–15). Based on these preliminary results, we seeded Rh30 and RD cells, transfected them with NS and SSO55 and 24 h post-transfection used the IGF-1R targeted antibody (MK0646) to block the IGF-1R/IGF-2 pathway^49^. In the presence of complete media, the addition of SSO55 decreased the basal p-AKT levels (Fig. 5F, Supplementary Fig. 7B lanes 1 and 2). The antibody blockage almost completely abrogated the phosphorylation of AKT (Ser473) in both NS and SSO55 treated cells (Fig. 5F, Supplementary Fig. 7B lanes 3 and 4). However, tumor cells secrete IGF-2, and tumor-derived IGF-2 is persistent in the tumor microenvironment, keeping this signaling cascade activated. To mimic that microenvironment, we added recombinant IGF-2 to the cells in the presence of the IGF-1R antibody and probed again for AKT phosphorylation (Ser473). Interestingly, we found that in both Rh30 and RD cells the phosphorylation cascade is reactivated after just 2 min of IGF-2 activation. We harvested these cells after 2, 5, and 10 min of IGF-2 addition and found that in the combinatorial presence of an IGF-1R Ab and SSO55, the increase in p-AKT is significantly less than the IGF-1R Ab alone treated cells (Fig. 5F, Supplementary Fig. 7B lanes 5 and 6 (2 min), lanes 7 and 8 (5 min) and lanes 9 and 10 (10 min)). We repeated these experiments using the 10 min time-point and the quantified results are shown (Fig. 5G).

These data reinforce our earlier findings and strengthen the idea that the combinatorial treatment of SSO and IGF-1R blocking antibodies act synergistically to abrogate the mitogenic as well as angiogenic signaling of the IR/IGF pathway.

### SSO treatment attenuates IR signaling and reduces vessel formation in *SCID* mice

**Fig. 6:**
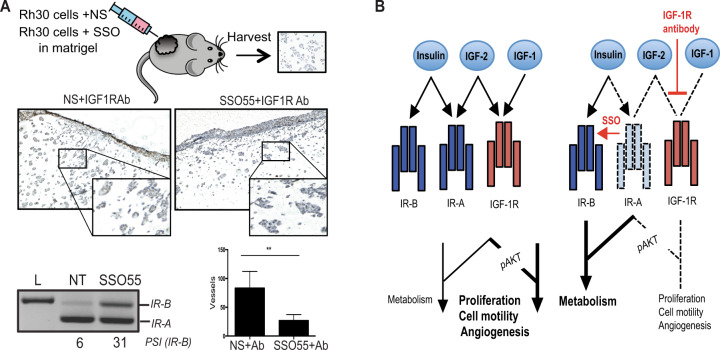
SSO treatment attenuates insulin receptor signaling and reduces vessel formation in *SCID* mice. **A** Mice were injected with 10^6^ Rh30 cells either transfected with NS or SSO55. IGF1-R antibody was administered on days 1 and 3 post-injection. The matrigel plugs were extracted after 7 days for CD34 (endothelial cell marker) staining. The plugs were formalin-fixed and paraffin-embedded, and slides were stained for CD34. Quantification of the CD34 staining from NS and SSO55-treated grafts was done in a blinded manner, *n* = 10 mice in each condition. The representative IHC pictures for CD34 staining are depicted. The RT-PCR shows the splicing changes in the injected cells in the presence of SSO55. **B** Model depicting how insulin and IGF-2 bind to *IR-A* in tumor cells leading to proliferative signaling while in the presence of an IGF-1R antibody and an SSO that shifts the splicing to IR-B, the proliferative signaling (AKT) can be mitigated. Results are shown as the standard error of the mean (±SEM).

In order to determine the ability of SSO55 to interfere with angiogenesis in vivo, we used the well-characterized Matrigel Plug Angiogenesis Assay^56^. Briefly, we injected 10^6^ RMS Rh30 and RMS RD cells embedded in Matrigel that had been transfected with either NS SSO or SSO55 24 h prior to implantation into flanks of nude mice. On days 1 and 3 post-injection, half of the mice were treated with anti-IGF-1R antibody (MK-0646). We harvested the plugs at day 7 and quantified the blood vessels formed in these Matrigel plugs by staining the paraffin sections for CD34/CD31 both of which are endothelial cell markers. We found that the tumors with SSO55 and IGF-1R Ab showed significantly less formation of blood vessels compared to IGF-1R Ab alone. To confirm the activity of the SSO we isolated RNA from the cells prior to injecting them into mice and found via RT-PCR that the percentage of the *IR-B* isoform was higher in the tumors that were transfected with the SSO55 (Fig. 6A, Supplementary Fig. 8).

Our data show that SSO55 can effectively shift the splicing of IR to *IR-B* isoform and consequently decrease angiogenesis in grafted tumor cells in the presence of IGF-1R antibodies. Therefore, SSOs that promote IR-B arise as a potentially viable therapeutic strategy either alone or in combination with existing IGF-2 therapies for RMS patients.

## Discussion

Our data show that IR is alternatively spliced to produce an exon 11-skipped isoform *IR-A* in RMS patient samples, cancer cells, as well as in stress conditions like hypoxia. We designed an SSO that blocks the CUG-BP1 binding site and can restore the splicing back to the full-length IR, *IR-B*. This modification in the alternative splicing of IR decreased proliferation and migration, (mitogenic as well as angiogenic potential) of RMS cell lines. Furthermore, we used an *IR-A* plasmid which is resistant to the splicing change by SSO55 and found that the overexpression of *IR-A* reinstates the migration and angiogenic capability of RMS cell lines in the presence of SSO55, showing that the effect of SSO55 is specific to the shift in *IR* splicing to *IR-B*. Additionally, when we injected the SSO55-treated cells into the flanks of *SCID* mice, we found decreased blood vessels as compared to the grafts treated with control SSO. Our data outline SSO55 induced inhibition of cancer hallmarks, a future therapeutic strategy for using *IR-B* promoting SSOs in combination with existing IGF-2 therapies for the treatment of RMS patients (Fig. 6B).

Additionally, our data link the RNA binding proteins CUG-BP1 and MBNL1 with IR alternative splicing and this interplay between the two could further be exploited in therapeutic and mechanistic directions for other cancers and diseases for which insulin signaling is affected. A recent paper documented the role of CUG-BP1 in breast cancer cells and showed that knockdown or overexpression of CUG-BP1 directly correlates to expression level changes in IR-A in these cells. The authors also queried breast cancer databases and found that CUG-BP1 levels are significantly increased in multiple types of breast tumor samples as compared to control normal tissue. Additionally, overexpression of CUG-BP1 increases the oncogenic potential of breast cancer cells shown by colony-forming asays^24^. This data strengthens the importance of our findings that CUG-BP1 is a critical RNA binding factor that binds to the IR pre-mRNA and increases the expression of IR-A. However, alternative splicing is a highly complex and dynamic cellular process requiring a multitude of molecular interventions. While our data endorse the function of CUG-BP1 and MBNL1 RNA binding proteins in hypoxic stress, there could be additional splicing regulators that play a major role in this splicing change in other contexts.

Overall, the somatic mutation burden is very low in RMS tumors as compared to other tumor types^57^. Therefore, in general, it is difficult to treat RMS by targeting the driver mutation. One of the rare classes of mutations in ERMS tumors are mutations in the RAS pathway, nevertheless, Shern et al. show that while 9/43 Rhabdomyosarcomas have a *RAS* mutation, targeting the RAS pathway either alone or in combination demonstrated no detectable anti-RMS cytotoxicity^57,58^. In addition to this, the most common mutations in RMS tumors were found to be in the receptor tyrosine kinase RAS/PIK3CA axis^57^. Because the IR signaling culminates in the activation of the PI3-kinase pathway, and because our data demonstrated potent inhibition of AKT phosphorylation in an IGF-2 replete tumor microenvironment, it is worthwhile to attenuate the signaling in this pathway by targeting the *IR* alternative splicing in combination with other treatment strategies.

Taken together, our data unveil a new paradigm of alternative splicing regulating cellular behavior to mediate cancer-causing changes and how SSO compounds can reinstate normal splicing and impede tumor growth. Splice switching technology has been successfully employed and is an FDA-approved clinical treatment in SMA patients, giving credence for clinical applications of SSO technology^41^. The target specificity of SSOs still remains a challenge and needs to be vetted carefully for off-target effects. Given the inadequacy as well as the toxic side effects of chemotherapy in pediatric patients with advanced cancer, it is a crucial time to devise novel, out-of-the-box therapy options, to not only modulate the signaling pathways more ingeniously but also to open up new areas of therapeutics. In this work, we provide insight into alternative splicing changes and the consequential aberrant downstream signaling during tumorigenesis; moreover, our SSO compounds propose innovative RMS therapeutics that could be potentially translatable to the treatment of other types of cancer associated with aberrant alternative splicing.

## Methods

### RMS patient samples

Human tissue samples were obtained from the Cooperative Human Tissue Network, Pediatric Division at Columbus Nationwide Children’s Hospital after institutional review board approval. All specimens were snap-frozen and stored at −80 °C. The tissue was ground using a mortar and pestle in liquid nitrogen. RNA was extracted from tissue samples (25–40 mg) using RNeasy Mini Protocol (Qiagen, Cat # 74106). Typically 1 µg of RNA was used for RT in 20-µl reactions^28^. This study received the institutional review board (IRB) approval from the Abigail Wexner Research Institute, Nationwide Children’s Hospital and The Ohio State University.

### Plasmids and minigenes

The *IR* 10-11-12 minigene was a kind gift from Dr. Nick Webster. The INSR-A GFP expression plasmid was constructed from the INSR-B GFP plasmid obtained from Addgene (Plasmid #22286). Exon 11 was deleted from the cDNA construct and silent mutations were introduced in exon 12 using QuikChange Lightning^™^ mutagenesis kit (Agilent, cat# 210519), using the following mutagenic primers: FW 5′GCCGGAGATGACCAGGCTCTCTTTATTCACCACCTTCTCAAAAGGCCTG3′ and Rev 5′CAGGCCTTTTGAGAAGGTGGTGAATAAAGAGAGCCTGGTCATCTCCGGC3′.

### RT and PCRs

RT reactions were carried out using 1 μg of RNA using Transcriptor RT enzyme (Catalog No. 03531287001) from Sigma Aldrich. *IR* minigene PCRs were performed as previously reported. PCRs for endogenous *IR* was performed using a primer on exon 10 5′GGCTGAAGCTGCCCTCGAG3′ and a primer on exon 12 5′GCGACTCCTTGTTCACCACC3′. This PCR generates a larger full-length product (160 bp) and a smaller exon-11-skipped product (135 bp). The amplicons were amplified using the following primers under the standard PCR conditions: (94 °C 5′, 35 cycles of 94 °C 30″, 65 °C 30″, 72 °C 1′, 72 °C 7′). The IR-A GFP construct expression was detected using the same forward primer in exon 10 together with a primer carrying complementary mutations in exon 12 (5′GGCTCTCTTTATTCACCAC3′) and the same PCR conditions described previously.

### Western blot analysis and antibodies

Cells lysed in NP-40 buffer and equal amounts of protein were loaded in sodium dodecyl sulfate sample buffer onto a sodium dodecyl sulfate-polyacrylamide gel, blotted onto a polyvinylidene difluoride membrane, and analyzed for expression of either MMP-9 (1:500; R&D Systems AF911), p-AKT (1:1000; Cell Signaling 4060), pan AKT (1:1000; Cell Signaling 4691), GAPDH (1:7000; Cell Signaling 2118), INSR (1:500; Cell Signaling 74118), and Hif-1α (1:250; BD Biosciences Catalog number 610959). To detect the expression of β-Actin clone AC-15 (1:2000; Catalog Number A5441) from Sigma was used. Protein sizes were determined using the Precision Plus Protein Dual Color Standards marker (Catalog Number 161-0374) from Life Technologies (Carlsbad, CA, USA). The IGF-1R Ab (0.05 µg/µl final concentration) used was MK-0646 obtained was a kind gift from the PPTC^59^. The recombinant human IGF1 and IGF2 were purchased from R&D biosystems (Cat. Number 291-G1 and 292-G2) (10 ng/ml final concentration). All blots were derived from the same experiment and were processed in parallel.

### Quantitative migration assay

Rh30 cells were transfected with 100 nM SSO55 or 100 nM NS were placed in the top chamber of a dual-chamber Boyden assay. Non-transfected Rh30 cells were used as a control. Cells were exposed to an fetal bovine serum (FBS) gradient (2% upper chamber and 15% lower chamber) or no gradient as a control (2% FBS in both chambers). Percent migration was quantified by dividing the number of cells that migrated through the porous membrane by the total amount of cells (cell amount gauged by crystal violet staining and subsequent optical density measurement at 570 nm).

### Proteome profiler microarray

The protein profiler Array^TM^ Human Angiogenesis kit array kit was purchased from R&D Biosystems. Briefly, antibodies have been spotted in duplicate on nitrocellulose membranes. Cell lysates treated with NS, or SSO55, with and without IGF-1R-blocking antibodies were mixed with a cocktail of biotinylated detection antibodies. The sample/antibody mixture was then incubated with the Human Angiogenesis Array. Any protein/detection antibody complex present was bound by its cognate immobilized capture antibody on the membrane. Following a wash to remove the unbound material, streptavidin–HRP and chemiluminescent detection reagents are added sequentially. Light is produced at each spot, proportional to the amount of analyte bound.

### Angiogenesis co-culture experiment

We transfected Rh30 and RD cells with NS and SSO55. After 24 h the cells were treated with the IGF-1R antibody. After another 24 h, the HUVEC cells were plated at the matrigel coated the bottom with media from transfected Rh30 or RD cells. The vascular structures (tubes) in the endothelial cells were quantified using the CD34 staining^60^.

### Matrigel plug angiogenesis assay

Rh30 cells transfected with NS or SSO55 were resuspended in matrigel and injected into flanks of nude mice. Ten mice were used for each NS and SSO55 and 2 million cells were injected into each mouse flank. On days 1 and 3 post-injection, 5/10 mice in each group were treated with IGF-1R antibody (MK0646). Matrigel tumors were excised after 7 days, cross-sections taken, stained with CD34 in a blinded fashion, and counterstained with Meyers stain. All mouse experiments were done following the institutional International Animal **C**are and **U**se **C**ommittee (IACUC) policies and guidelines (IACUC protocol number: AR15-00119).

### RNA electrophoretic mobility shift assay

The INSR I10 probe was in vitro transcribed from a PCR product amplified from human genomic DNA (forward primer 5′-TAATACGACTCACTATAGGGAGCGAGTGGTCCAGGGTCAAA-3′; reverse primer 5′-AAAACCAACGCCTTTGAGGACAGA-3′) using the MEGAshortscript™ T7 Transcription Kit (Thermo Fisher Scientific, Invitrogen™ Catalog number AM1354) and Biotin-14-CTP (Thermo Fisher Scientific, Invitrogen™ catalog number 19519-016) following the manufacturer instructions. For the REMSA the LightShift™ Chemiluminescent RNA EMSA Kit (Thermo Fisher Scientific, Pierce™ catalog number 20158) was used following manufacturer instructions. Briefly, reactions containing 1 fmol of the biotinylated INSR I10 probe and 400 nM of recombinant GST-CUGBP1 (Novus Biologicals, Abnova™ catalog number H00010658-P01-2ug) were incubated for 25 min at room temperature together with increasing concentrations of either a non-specific SSO or SSO55 (0, 50, 100, and 250 nM) and ran on a 5% TBE polyacrylamide gel for 75 min at 120 V, then transferred to a nylon membrane (Thermo Fisher Scientific, Biodyne™ Catalog number 77016). Complexes were detected by chemiluminescence.

### Quantification of splicing ratios

Percentages of full-length and exon-excluded products were quantitated using Image-Quant TL (Version 8.1). The significance of the results was assessed using the two-tailed Student’s *t* test using GraphPad Prism (Version 6.0). The percent expression of IR-B is represented as PSI.

### Cell culture, growth, and transfection conditions

RMS cell lines (Rh30, RD, SMS-CTR) cells were obtained from PPTC^59^. HeLa and HeLa S3 cells were obtained from ATCC (CCL-2 and CCL-2.2). HUVEC was obtained from ATCC (CRL-1730). All cell lines derived from human material have been verified by STR analysis. Experiments were performed within the first 10 passages of thawing cells. RMS cells were maintained in DMEM, whereas HeLa S3 cells were maintained in the RMPI medium. Both were supplemented with 10% fetal bovine serum (Catalog Number SH3007103) from Thermo Fisher Scientific, 1× l-glutamine (Catalog Number MT 25-005 CI) from Corning, and 1× penicillin/streptomycin (Catalog Number MT 30-001 CI) by Corning. One microgram of each plasmid was transfected into cells using lipofectamine 3000 (Catalog number L3000015). For the IR-A GFP expression experiments an EGFP expression plasmid was used as a negative control.

### SSO treatment and siRNA knockdowns

SSOs were kindly provided by *Ionis Pharmaceuticals*. SSOs specific to IR pre-mRNA were transfected in cells with Lipofectamine 2000 (Catalog Number 11668030) from Life Technologies. Similarly, Hif-1α specific siRNA (siRNA ID s6541; Cat# 4390824) or a non-specific siRNA were transfected into cells with lipofectamine RNAiMAX (Life Technologies catalog number 13778075) and cells were treated with normoxia or hypoxia (1% O_2_) for 24 h before analysis. SSO sequences are listed in Supplementary Table 1.

### Statistical analyses

All statistical analyses were performed using *GraphPad prism for MacOS*

### Reporting summary

Further information on research design is available in the Nature Research Reporting Summary linked to this article.

## Supplementary information

Supplementary figures and tables

REPORTING SUMMARY

## Data Availability

All data generated or analyzed are included in the published article and its Supplementary Information files. Materials/reagents are available from the corresponding author on reasonable request.
