# Supplementary material for: Splice-switching of the insulin receptor pre-mRNA alleviates tumorigenic hallmarks in rhabdomyosarcoma
Source: NPJ Precis Oncol. 2022 Jan 11;6:1. doi: 10.1038/s41698-021-00245-5 (PMC8752779; doi:10.1038/s41698-021-00245-5)

SUPPLEMENTARY FIGURES AND TABLES

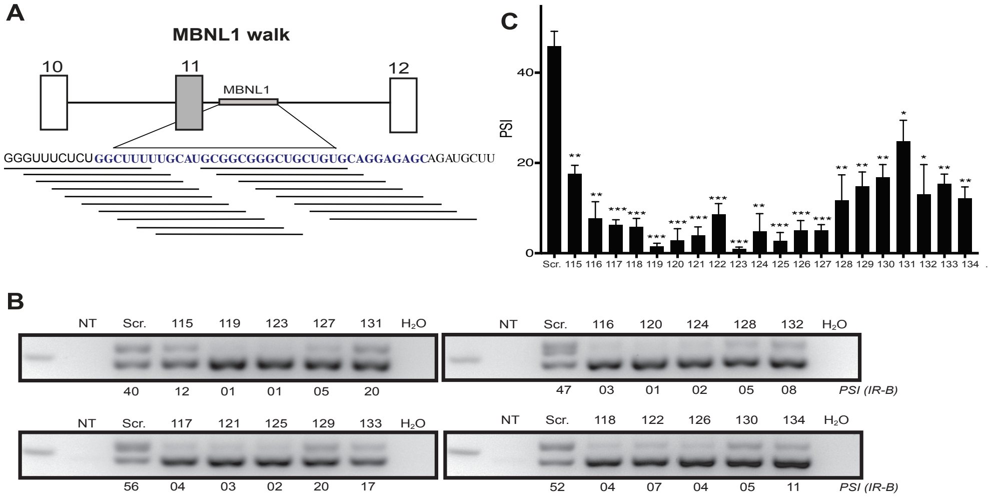

Supplementary Figure 1: **Splice switching oligonucleotide (SSO) walk for the MBNL binding site in intron 11 changes *IR* splicing to *IR-A*.**

A, B. Representation of the insulin receptor minigene. The *MBNL1* binding site is shown on the *IR* pre-mRNA. The black lines represent the SSOs used for the MBNL1 walk. RT-PCRs depicting the IR isoforms in the presence of control SSOs or SSOs that target the MBNL1 binding site are shown below the sequences. C. The quantification for n=3 experiments is shown. Results are shown as standard error of the mean (+/-SEM).

SUPPLEMENTARY FIGURES AND TABLES

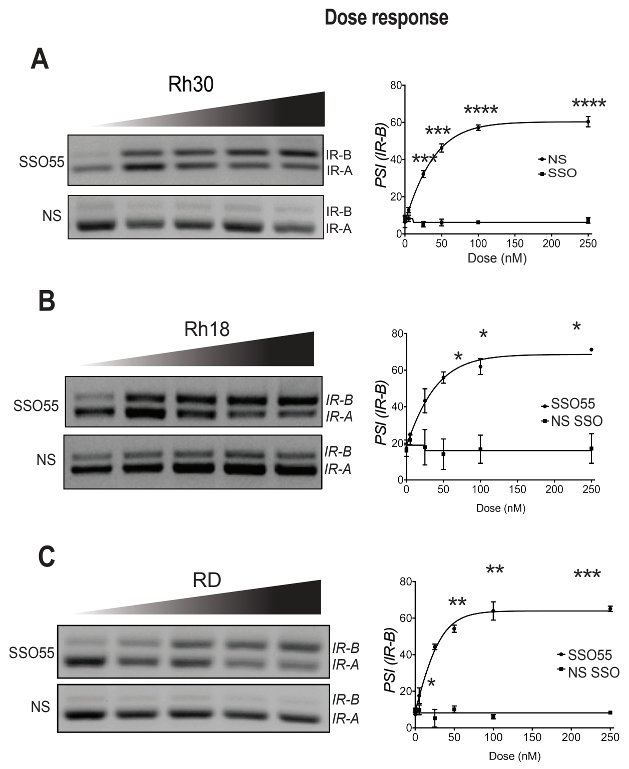

Supplementary Figure 2: **Dose response of RMS cell lines Rh30, Rd and Rh18 for SSO55.**

500000 Rh30, RD and Rh18 cells were seeded, transfected with different concentrations (0-20nm) of non-specific (NS) and SSO55. RNA was extracted after 24 hours and RT-PCR for insulin receptor was performed. Dose response curve of SSOs showing the IR isoforms and their quantification in three RMS cell lines is shown. Results are shown as standard error of the mean (+/-SEM).

SUPPLEMENTARY FIGURES AND TABLES

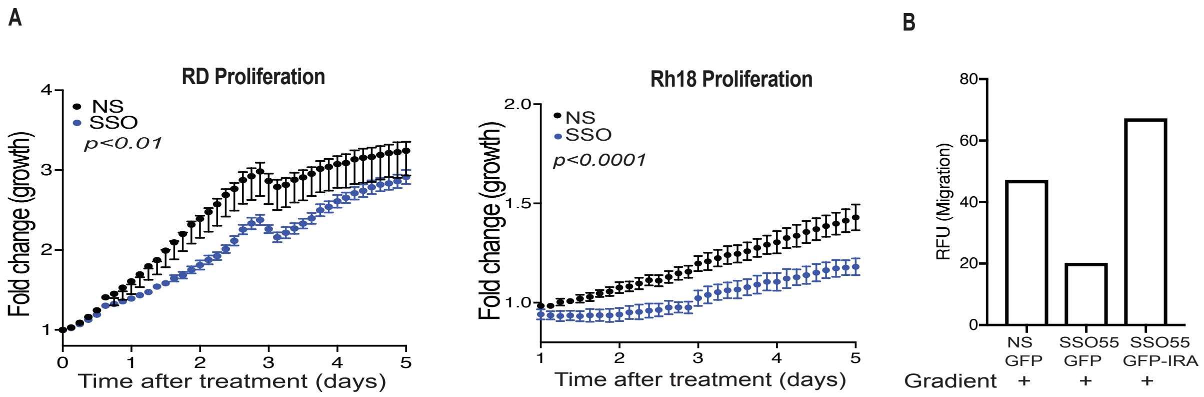

Supplementary Figure 3: SSO treatment decreases proliferation in RMS cell lines.

A RD and Rh18 cells were seeded, transfected with either nonspecific (NS) or SSO55 and subjected to proliferation assay using the Incucyte® software. B. Trans-well migration assay: Rh30 cells were transfected with GFP or GFP IR-A as well as NS or SSO55 and placed in the top insert of a dual-chamber 24 well plate and exposed to an FBS gradient. Number of cells migrating to the lower chamber was quantified. Results are shown as standard error of the mean (+/-SEM).

SUPPLEMENTARY FIGURES AND TABLES

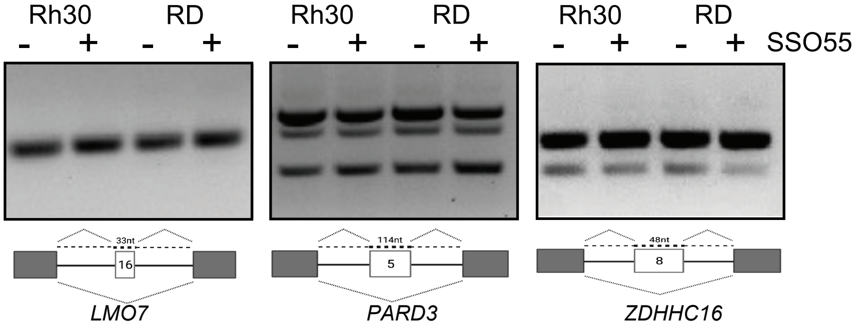

Supplementary Figure 4: SSO55 specifically binds to IR.

Rh30 or RD cells were transfected with SSO55 (+) or NS SSO (-) and RT-PCR analyses of representative genes which their alternative splicing is known to be regulated by the CUG-BP1 splicing factor (45) were performed.

SUPPLEMENTARY FIGURES AND TABLES

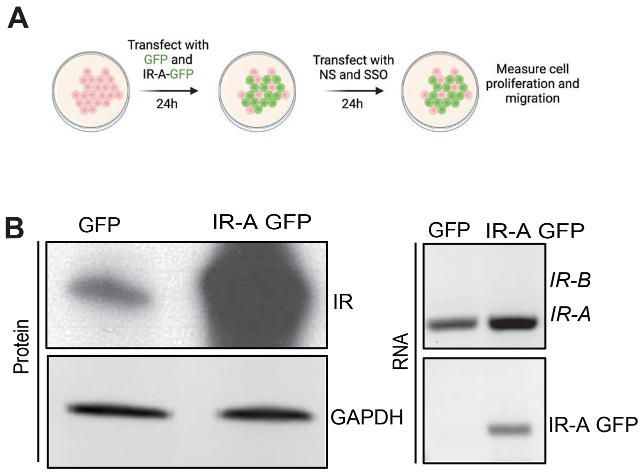

Supplementary Figure 5: The addition of a splice resistant *IR-A* plasmid rescues the functional effects of SSO55.

A. Schematic representation of the IR-A-GFP cDNA overexpression experiments performed to measure the effects on cell proliferation. **B**. Western blot and RT-PCR analyses of the IR-A-GFP overexpression experiments using GFP as a negative control. RT-PCR was performed with primers to detect both endogenous INSR, as well as with mutant primers to detect the IR-A-GFP cDNA construct.

SUPPLEMENTARY FIGURES AND TABLES

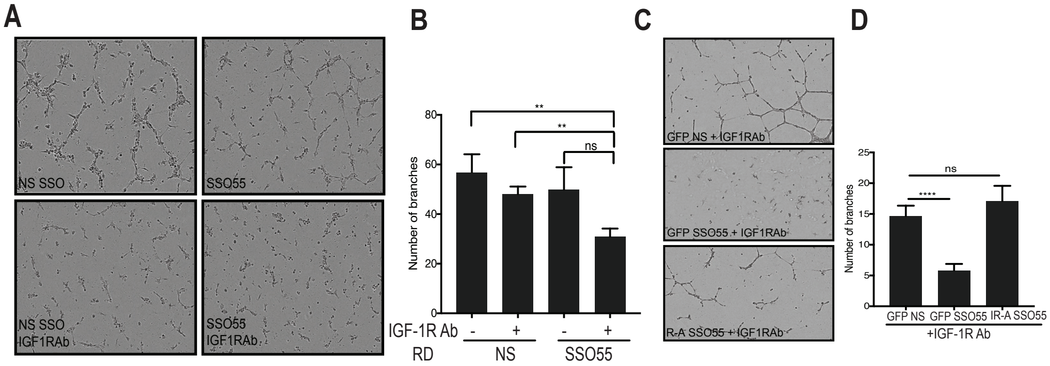

Supplementary Figure 6: SSO treatment diminishes tube formation in RMS cells.

A. Human Umbilical Vein endothelial cells (HUVEC) were plated on basement membrane matrix and incubated with either PBS or media from RD cells treated with either NS or SSO55 alone or in combination with IGF-1R antibody. **B**. The data is quantified; statistics for n=3 experiments, Unpaired T- test, *P value*= 0.0058, 0.0020 and 0.0683 (ns) respectively. **C**. Rh30 cells were seeded in a 96-well plate and, transfected with either IR-A-GFP or GFP and NS SSO or SSO55. IGFR Ab was added to all the wells. After 24 hours the media was collected and HUVECs were plated and incubated with media from treated Rh30 cells. The tubes were quantified using pictures from incucyte. **D**. The data is quantified, Paired T-test, P value <0.0001 and 0.416 (ns) respectively. Results are shown as standard error of the mean (+/-SEM).

SUPPLEMENTARY FIGURES AND TABLES

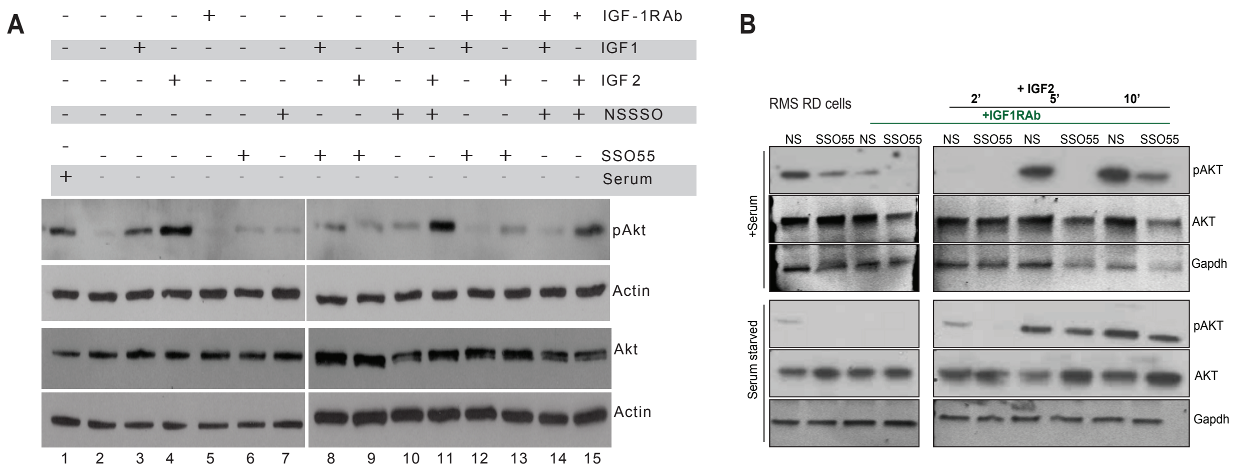

Supplementary Figure 7: **The additions of SSO55 attenuates the AKT signaling pathway**.

Rh30 cells were seeded, transfected with NS and SSO55, treated with IGF-1R Ab followed by the ligands IGF1 and IGF2 and blotted for pAKT, total AKT and Actin as shown. This experiment helped optimize the conditions for the experiment shown in the main Figure 5 and Figure S6C for Rh30 and RD cells. **B**. RD cells were seeded in a 6-well plate, transfected with NS or SSO55. After 24 hours, all wells but the controls were treated with IGF-1R antibody (depicted in green). After 30 min, one NS and SSO55 treated well was harvested and IGF2 was added to the remaining wells. The wells were harvested with RIPA buffer containing protease and phosphatase inhibitors after 2 min, 5 min and 10 min. The blots for phosphor AKT, total AKT and Gapdh are depicted. The data is shown for Serum containing and serum starved cells. Cells were starved for 6 hours before the addition of the IGF-1R Ab.

SUPPLEMENTARY FIGURES AND TABLES

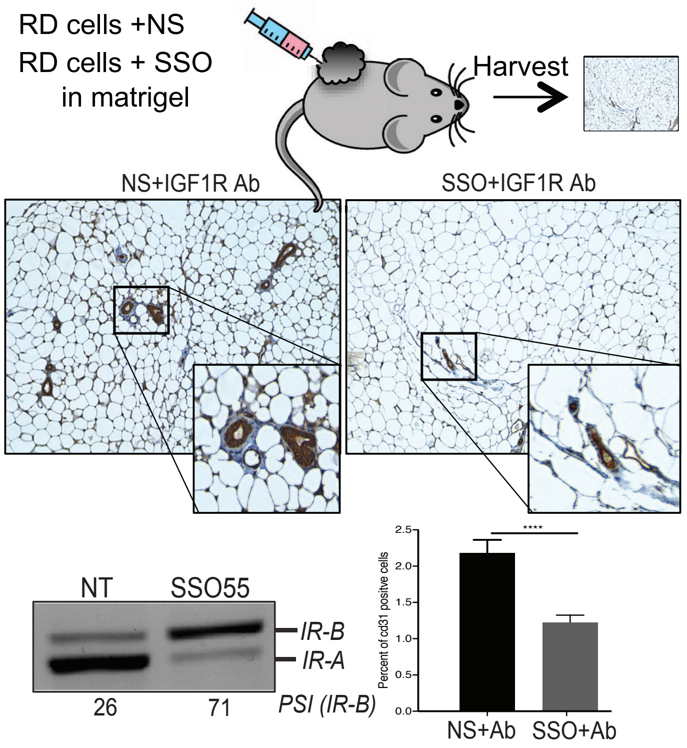

Supplementary Figure 8: SSO treatment attenuates insulin receptor signaling and reduces vessel formation in *SCID* mice.

D. Mice were injected with 10^6 RD cells either transfected with NS or SSO55. IGF1-R antibody was administered at day 1 and 3 post-injection. The matrigel plugs were extracted after 7 days for CD31 (endothelial cell marker) staining. The matrigel plugs were formalin fixed and paraffin embedded, and slides were stained for CD31. Quantification of the CD31 staining from NS and SSO55-treated grafts was done, n=6 samples each condition. The representative IHC pictures for CD31 staining are depicted. The RT-PCR shows the splicing changes in the injected cells in the presence of SSO55. Results are shown as standard error of the mean (+/-SEM).

SUPPLEMENTARY FIGURES AND TABLES

Ionis #	Sequence	Ionis #	Sequence
6167**50**	CCACATGTGTCCGAGTAA	6329**11**	CATTAGACAGACCACTGG
6167**51**	GGAGGCCACATGTGTCCG	6329**12**	TTCATTAGACAGACCACT
6167**52**	CACTTGGAGGCCACATGT	6329**13**	ACTTCATTAGACAGACCA
6167**53**	TCTGACACTTGGAGGCCA	6329**14**	GAACTTCATTAGACAGAC
6167**54**	TGGGCTCTGACACTTGGA	6329**15**	GGGAACTTCATTAGACAG
6167**55**	ACCACTGGGCTCTGACAC	6329**16**	GAGGGAACTTCATTAGAC
6250**81**	CTCTGACACTTGGAGGCC	6329**17**	GGACAGAGGGAACTTCAT
6250**82**	GCTCTGACACTTGGAGGC	6329**18**	TTTGAGGACAGAGGGAAC
6250**83**	GGCTCTGACACTTGGAGG	6250**89**	CGGCACCAGTGCCTGA
6250**84**	GGGCTCTGACACTTGGAG	6250**90**	CTCGGCACCAGTGCCTGA
6250**85**	CTGGGCTCTGACACTTGG	6250**91**	CGGCACCAGTGCCTG
6250**86**	ACTGGGCTCTGACACTTG	6250**92**	TCGGCACCAGTGCCTG
6250**87**	CACTGGGCTCTGACACTT	6250**93**	CGGCACCAGTGCCT
6250**88**	CCACTGGGCTCTGACACT	6250**94**	TCGGCACCAGTGCCT
6329**07**	AGACCACTGGGCTCTGAC	6250**95**	CTCGGCACCAGTGCCT
6329**08**	ACAGACCACTGGGCTCTG	5821**23**	CTGCCCGCCGCATGCAAA
6329**09**	AGACAGACCACTGGGCTC	4392**72**	TTAGTTTAATCACGCTCG
6329**10**	TTAGACAGACCACTGGGC		

Supplementary Table 1: Sequence of Splice switching nucleotides (SSOs).

SUPPLEMENTARY FIGURES AND TABLES

	Gene_ID	Gene Symbol	Accession	Mismatch	Mismatch	Position	Binding site location	CELF1 RIP?
	INSR	NC_000019.10_TRUNC_7112255_7294405_COMP	143804	0		18		YES!!!!
	GSTCD	NC_000004.12_TRUNC_105708778_105848023	52649	1	8:A>C	9	Intron 5 middle	no
	CDH4	NC_000020.11_TRUNC_61252426_61940617	654385	1	8:A>C	9	intron 8 last 2/3rd	yes
	SEPTIN9	NC_000017.11_TRUNC_77281410_77500596	48035	1	12:T>A	12	exon 12 3UTR	no
	POU6F2	NC_000007.14_TRUNC_38977878_39468601	429664	1	15:C>G	15	intron 6 first 1/3rd	no
	C7orf50	NC_000007.14_TRUNC_978485_1138291_COMP	86814	2	1:C>G,3:G>T	14	intron 2 middle	yes
	BAHCC1	NC_000017.11_TRUNC_81395431_81466332	13838	2	0:A>T,3:G>T	14	Intron 2 middle	yes
	SEPTIN9	NC_000017.11_TRUNC_77281410_77500596	217689	2	0:A>T,3:G>T	14		no
	CYMP	NC_000001.11_TRUNC_110480766_110491269	67	2	0:A>T,3:G>T	14		no
	LOC105371763	NC_000017.11_TRUNC_38691493_38704741	128	2	0:G>T,5:T>A	12		no
	EFNA1	NC_000001.11_TRUNC_155127873_155134910	5701	2	0:G>T,5:T>A	12		no
	ZNF423	NC_000016.10_TRUNC_49487531_49857919_COMP	153542	2	0:G>T,5:T>A	12		no
	RALGDS	NC_000009.12_TRUNC_133097720_133149220_COMP	36644	2	3:G>T,5:T>A	12	Intron 2 middle	yes
	CASZ1	NC_000001.11_TRUNC_10636602_10796676_COMP	20149	2	4:C>G,5:T>A	12		no
	ZNF180	NC_000019.10_TRUNC_44474428_44500539_COMP	5092	2	0:C>T,3:C>T	14	intron 2 first 1/3rd	yes
	CDH4	NC_000020.11_TRUNC_61252426_61940617	654202	2	2:A>G,3:C>T	14	intron 2 middle	yes
	CDH4	NC_000020.11_TRUNC_61252426_61940617	654294	2	2:A>G,3:C>T	14	intron 2 last 2/3rd	yes
	ACCSL	NC_000011.10_TRUNC_43920987_44060667	2793	2	0:A>T,7:A>C	10		no
	KSR2	NC_000012.12_TRUNC_117453012_117968558_COMP	368151	2	0:C>T,7:A>C	10		no
	NPAS3	NC_000014.9_TRUNC_32934785_33804176	590728	2	1:C>G,7:A>C	10		no
	SH2D3C	NC_000009.12_TRUNC_127738317_127778769_COMP	29339	2	6:G>C,7:A>C	10		no
	SLC37A4	NC_000011.10_TRUNC_119024351_119030906_COMP	4755	2	3:G>T,8:T>C	9		no
	WRB-SH3BGR	NC_000021.9_TRUNC_39380244_39515506	8270	2	1:A>G,8:G>C	9		no
	WRB	NC_000021.9_TRUNC_39380287_39397889	8227	2	1:A>G,8:G>C	9		no
	KCNH2	NC_000007.14_TRUNC_150944956_150978314_COMP	16219	2	3:A>T,8:G>C	9		no
	SERAC1	NC_000006.12_TRUNC_158109504_158168280_COMP	27530	2	3:A>T,4:T>G	13		no
	ANKRD52	NC_000012.12_TRUNC_56237807_56258391_COMP	17526	2	2:T>G,5:C>A	12	exon 28 3UTR	yes
	EMC8	NC_000016.10_TRUNC_85778624_85799744_COMP	3879	2	3:G>T,4:C>G	13		no
	MINDY4	NC_000007.14_TRUNC_30771417_30892387	13493	2	14:G>T,17:A>G	14		no
	INMT-MINDY4	NC_000007.14_TRUNC_30752135_30892387	32775	2	14:G>T,17:A>G	14		no
	KLF16	NC_000019.10_TRUNC_1852399_1863565_COMP	6883	2	10:G>A,17:C>G	10		no
	LOC105373831	NC_000002.12_TRUNC_198492130_198772330_COMP	61616	2	10:G>A,17:A>G	10		no
	CAPZB	NC_000001.11_TRUNC_19338773_19485641_COMP	142423	2	13:T>C,16:G>T	13	intron 8 first 1/3rd	yes
	PIEZO1	NC_000016.10_TRUNC_88715338_88785220_COMP	26513	2	14:C>T,16:G>T	14		no
	DHX32	NC_000010.11_TRUNC_125836337_125896483_COMP	16307	2	10:T>A,15:T>G	10		no

SUPPLEMENTARY FIGURES AND TABLES

	LOC105372693	NC_000020.11_TRUNC_57985899_57999562_COMP	7775	2	10:C>A,13:G>C	10		no
	GPATCH2	NC_000001.11_TRUNC_217426992_217631312_COMP	106005	2	9:T>G,11:T>G	9	intron 5 last 2/3rd	yes
	BEND2	NC_000023.11_TRUNC_18162931_18220904_COMP	14991	2	11:T>G,14:C>T	11		no
	ABCB1	NC_000007.14_TRUNC_87503863_87713323_COMP	128558	2	9:A>G,10:T>A	9		no
	LOC101929550	NC_000008.11_TRUNC_35672163_35710320_COMP	28607	2	6:G>C,17:C>G	10		no
	CUEDC1	NC_000017.11_TRUNC_57861243_57955323_COMP	88568	2	7:T>C,15:C>G	7		no
	C4orf51	NC_000004.12_TRUNC_145680103_145792248	74300	2	7:T>C,17:T>G	9		no
	LOC107984026	NC_000010.11_TRUNC_46335552_46838268	179167	2	3:A>T,17:A>G	13		no
	SCRN1	NC_000007.14_TRUNC_29920103_29990289_COMP	61248	2	3:A>T,17:C>G	13	intron 1 middle	yes
	ISOC2	NC_000019.10_TRUNC_55452978_55461682_COMP	7582	2	3:A>T,16:A>T	12		no
	ELP2	NC_000018.10_TRUNC_36129874_36177931	23687	2	3:A>T,9:T>G	8		no
	NOL4L	NC_000020.11_TRUNC_32443059_32585073_COMP	7684	2	3:A>T,9:T>G	8		no
	IGF2BP3	NC_000007.14_TRUNC_23310209_23470674_COMP	142955	2	1:T>G,17:C>G	15	intron 3 middle	yes
	1-Sep	NC_000016.10_TRUNC_30378133_30382850_COMP	743	2	8:G>C,14:C>T	8		no
	SLIT2	NC_000004.12_TRUNC_20251905_20620561	12764	2	8:G>C,15:T>G	8		no
	SLC24A2	NC_000009.12_TRUNC_19507452_20307888_COMP	221360	2	8:G>C,17:C>G	8		no
	LOC107986743	NC_000007.14_TRUNC_80245048_80249738_COMP	3153	2	8:G>C,11:C>G	8		no
	LOC105372018	NC_000018.10_TRUNC_22242790_22267817_COMP	3110	2	8:G>C,13:G>C	8		no
	TPD52L2	NC_000020.11_TRUNC_63865228_63891545	14121	2	8:G>C,10:T>A	8		no
	LARGE1	NC_000022.11_TRUNC_33162237_33922841_COMP	512057	2	1:C>G,17:A>G	15		no
	FCAMR	NC_000001.11_TRUNC_206957955_206970657_COMP	10559	2	1:C>G,14:G>T	12		no
	FCRL4	NC_000001.11_TRUNC_157573749_157598080_COMP	17368	2	1:C>G,16:A>T	14		no
	CLASP1	NC_000002.12_TRUNC_121337776_121649476_COMP	83443	2	1:C>G,15:C>G	13	intron 2 middle	yes
	LINC01138	NC_000001.11_TRUNC_148432959_148459920_COMP	6903	2	1:C>G,12:T>A	10		no
	LOC105371224	NC_000001.11_TRUNC_148442576_148461504_COMP	8487	2	1:C>G,12:T>A	10		no
	AGRN	NC_000001.11_TRUNC_1020102_1056119	17650	2	1:C>G,12:T>A	10		no
	RB1	NC_000013.11_TRUNC_48303747_48481890	140327	2	4:C>G,9:T>G	8		no
	UBAP1	NC_000009.12_TRUNC_34179005_34252523	22752	2	4:C>G,9:T>G	8	intron 4 middle	yes
	AMD1	NC_000006.12_TRUNC_110814621_110895713	25686	2	3:G>T,9:T>G	8	intron 4 first 3rd	yes
	TBX4	NC_000017.11_TRUNC_61452418_61485110	8596	2	0:C>T,16:C>T	15	intron 3 first 3rd	yes
	LOC107987163	NC_000011.10_TRUNC_77523814_77616414_COMP	55230	2	0:C>T,15:C>G	14		no
	LOC105371399	NC_000016.10_TRUNC_87766256_87775389	6286	2	0:C>T,15:C>G	14		no
	SLC39A9	NC_000014.9_TRUNC_69398379_69462390	14205	2	0:C>T,15:A>G	14		no
	OBSCN	NC_000001.11_TRUNC_228208013_228381431	109371	2	0:C>T,15:A>G	14	intron 10 last 2/3rd	yes
	SSC5D	NC_000019.10_TRUNC_55488503_55519099	7720	2	0:A>T,13:G>C	12		no
	LRP1B	NC_000002.12_TRUNC_140231423_142132463_COMP	526822	2	0:A>T,14:G>T	13	intron 76 middle	yes
	TONSL	NC_000008.11_TRUNC_144428780_144444488_COMP	8787	2	0:G>T,15:C>G	14		no
	ATP6V0A4	NC_000007.14_TRUNC_138706294_138799839_COMP	49229	2	0:G>T,9:T>G	8		no
	QRFPR	NC_000004.12_TRUNC_121328642_121381029_COMP	48490	2	5:T>A,9:A>G	8		no
	ZFR2	NC_000019.10_TRUNC_3804024_3869029_COMP	14923	2	7:A>C,10:G>A	7		no

SUPPLEMENTARY FIGURES AND TABLES

	LHPP	NC_000010.11_TRUNC_124461772_124614141	104344	2	5:G>A,14:G>T	8	intron 6 last 2/3rd	yes
	C16orf91	NC_000016.10_TRUNC_1419744_1420800_COMP	490	2	1:A>G,14:C>T	12		no
	HHIPL2	NC_000001.11_TRUNC_222501521_222548102_COMP	6772	2	1:A>G,14:C>T	12		no
	IGFBP7	NC_000004.12_TRUNC_57031071_57110385_COMP	7011	2	1:A>G,14:A>T	12	intron 1 middle	yes
	TSNAX-DISC1	NC_000001.11_TRUNC_231528653_232041272	435992	2	2:A>G,14:A>T	11		no
	DISC1	NC_000001.11_TRUNC_231626815_232041272	337830	2	2:A>G,14:A>T	11		no

Supplementary Table 2: Bowtie analysis of SSO55 sequence against human transcriptome.

Whole Blot scans of main and supplementary figures

Figure 2B

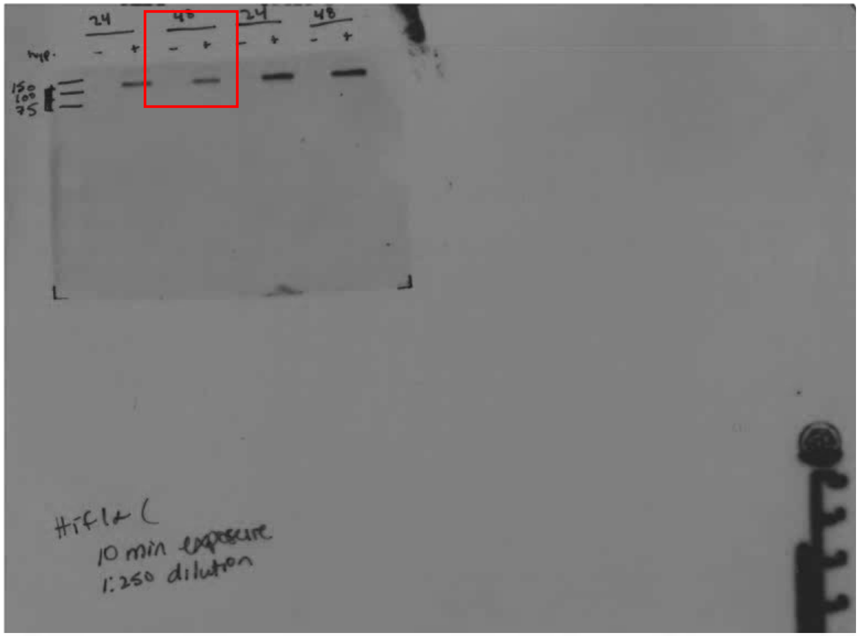

Hif1a

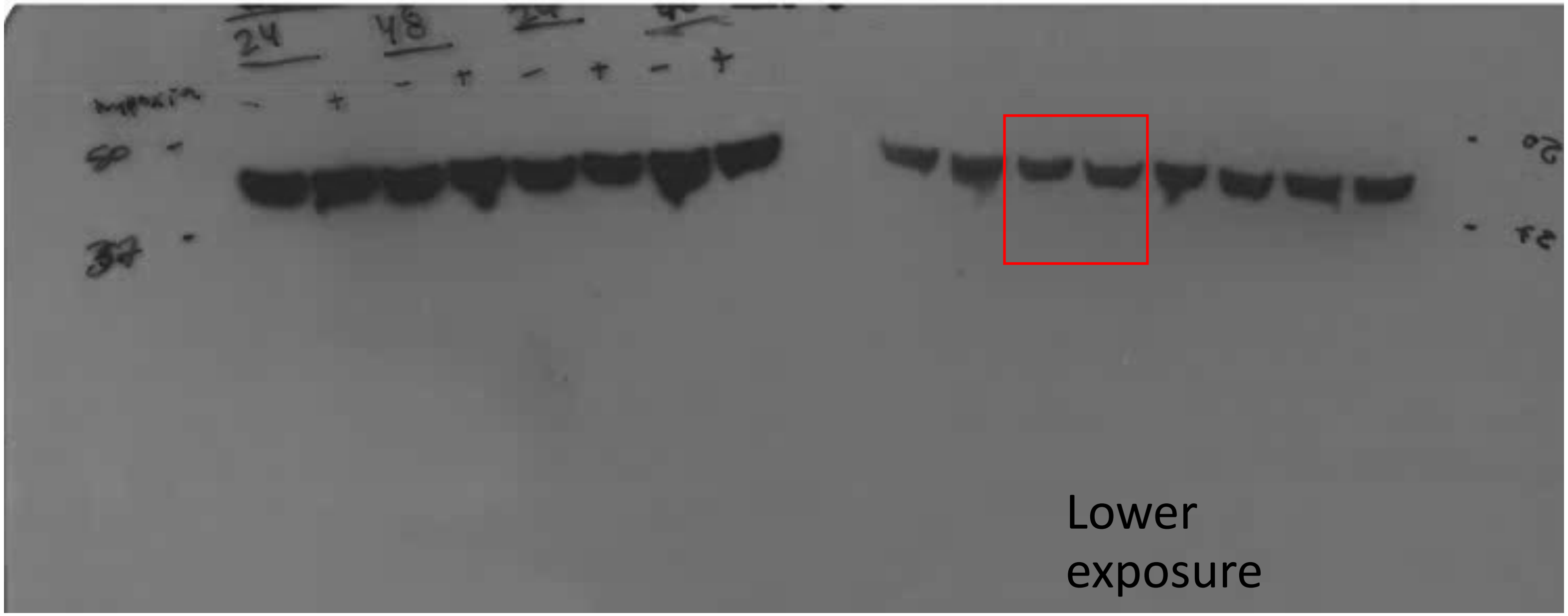

Beta Actin

Figure 2D

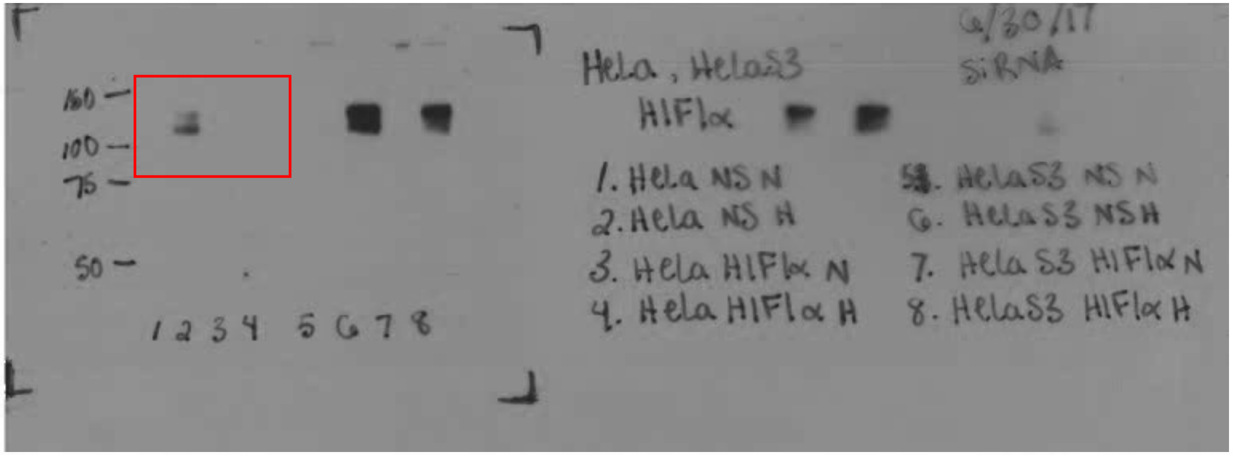

Hif1a

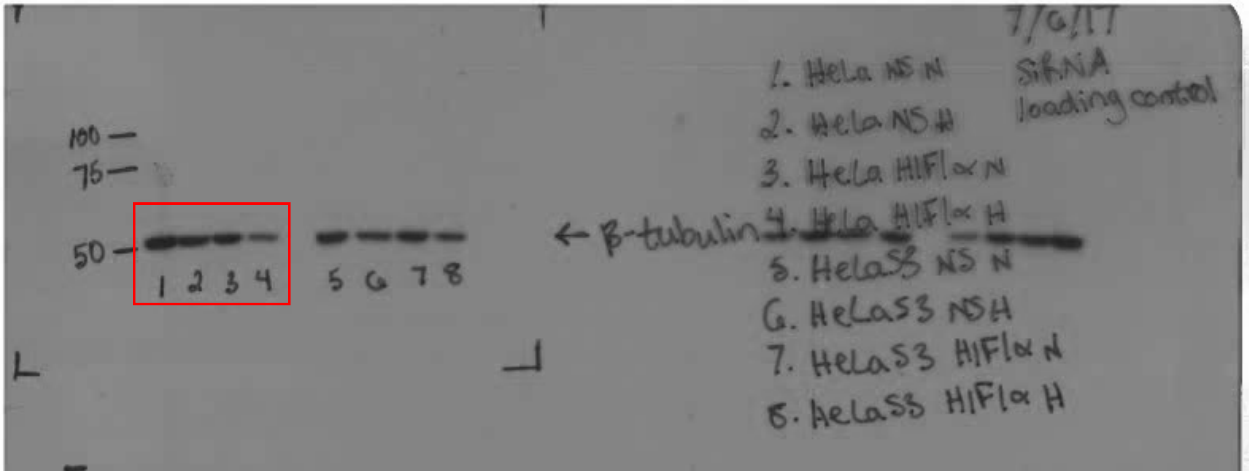

Beta Tubulin

Figure 3D

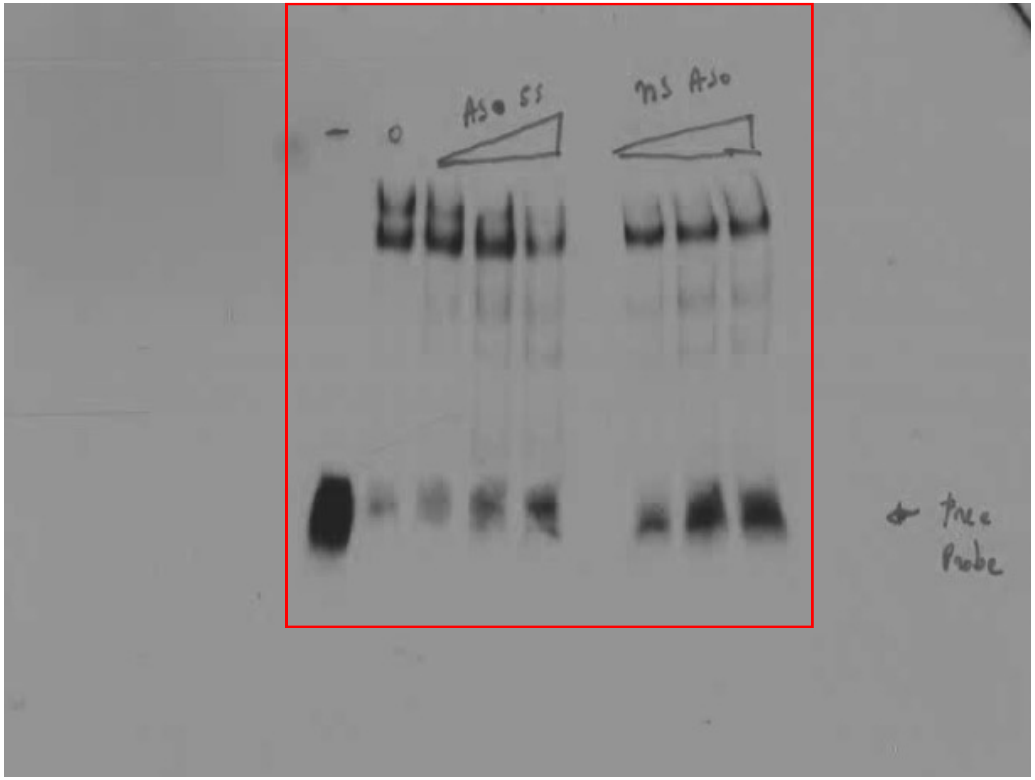

CUG-BP1 EMSA Blot

Figure 5C

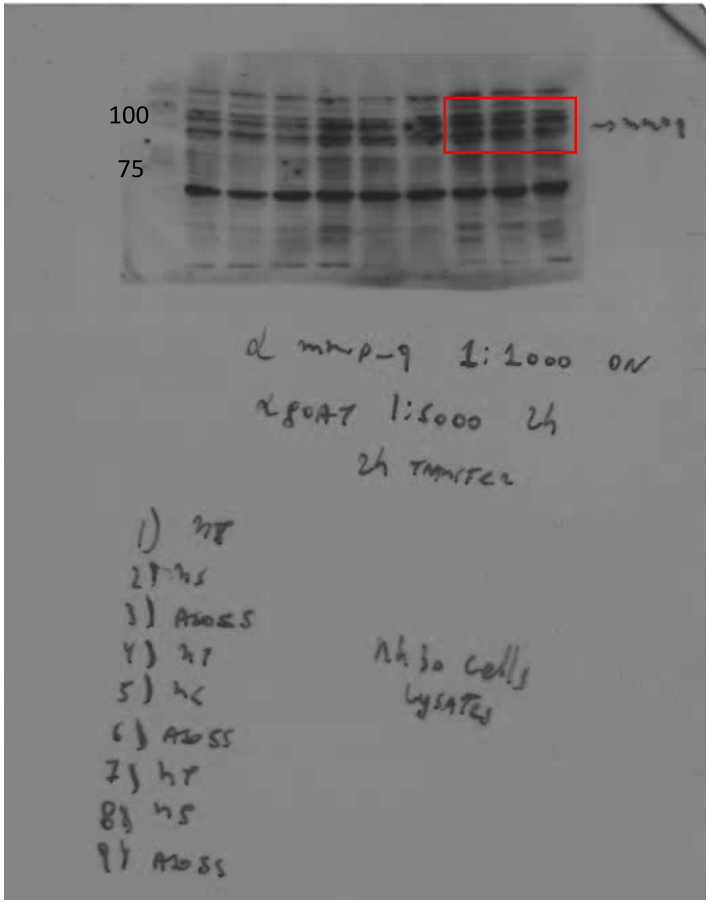

MMP-9

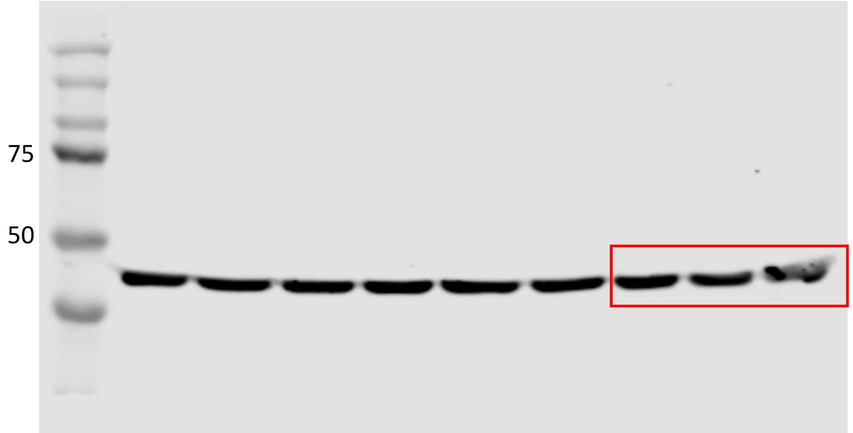

Actin

Figure 5F

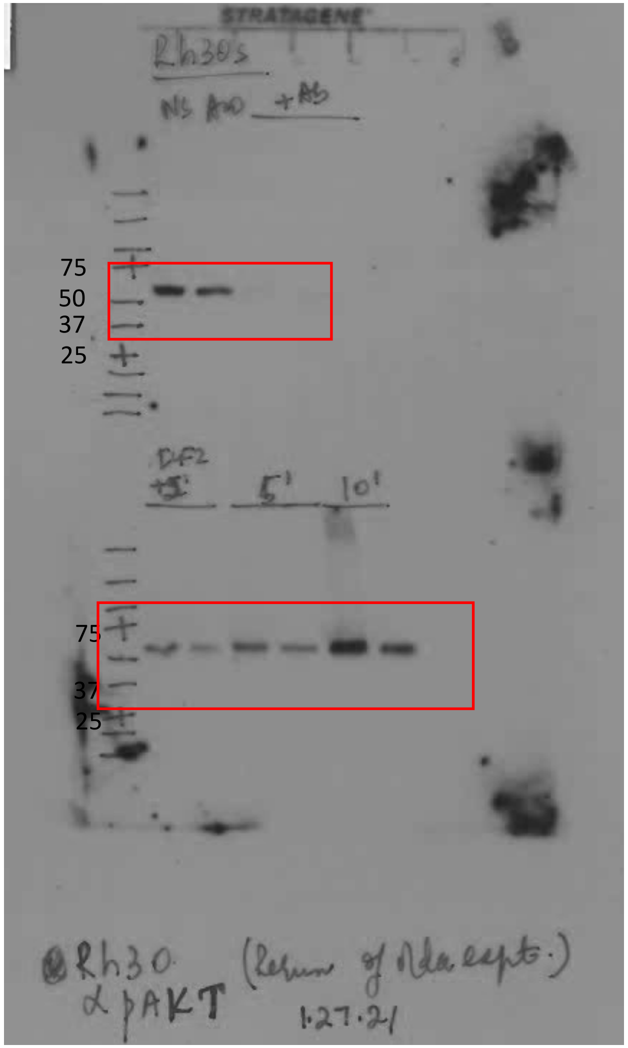

Rh30 pAKT

Figure 5F

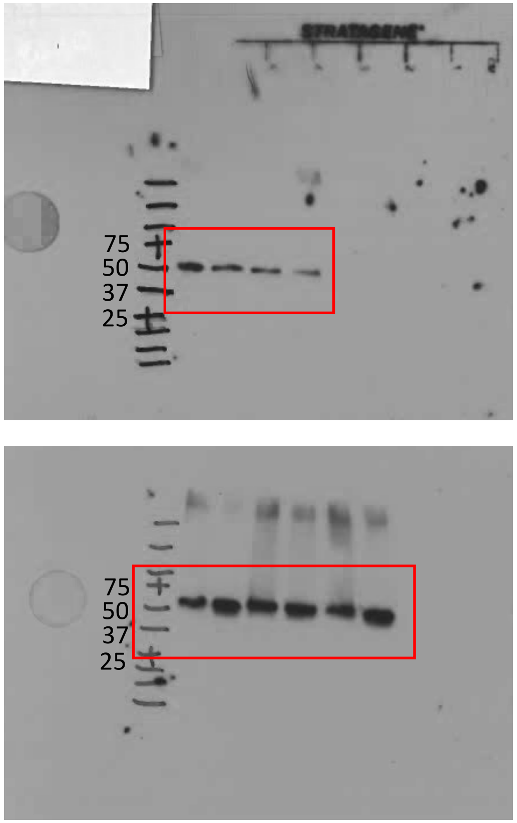

Rh30 AKT

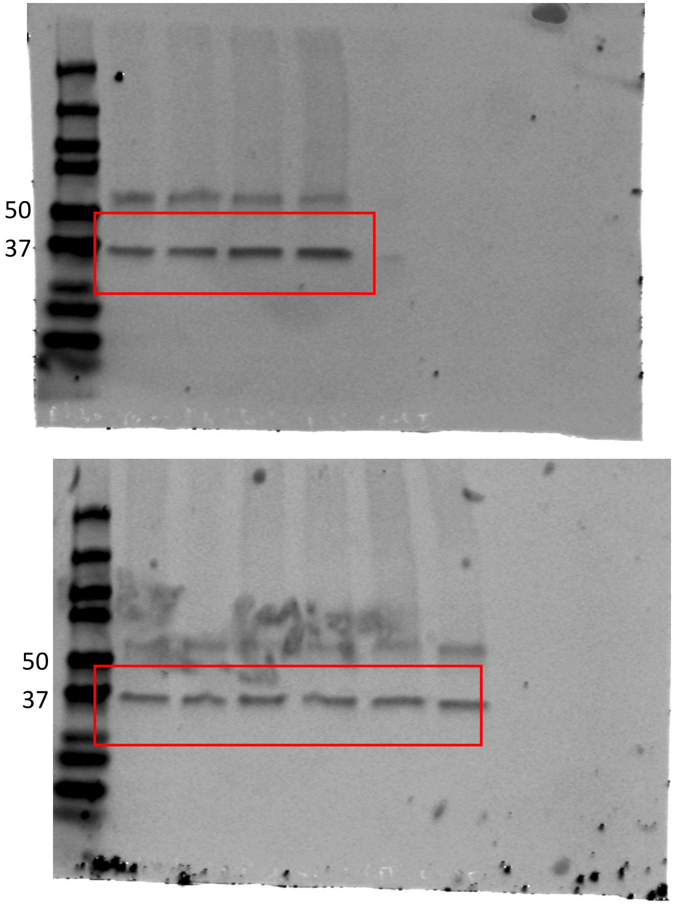

Rh30 Gapdh

Supplementary Figure 5

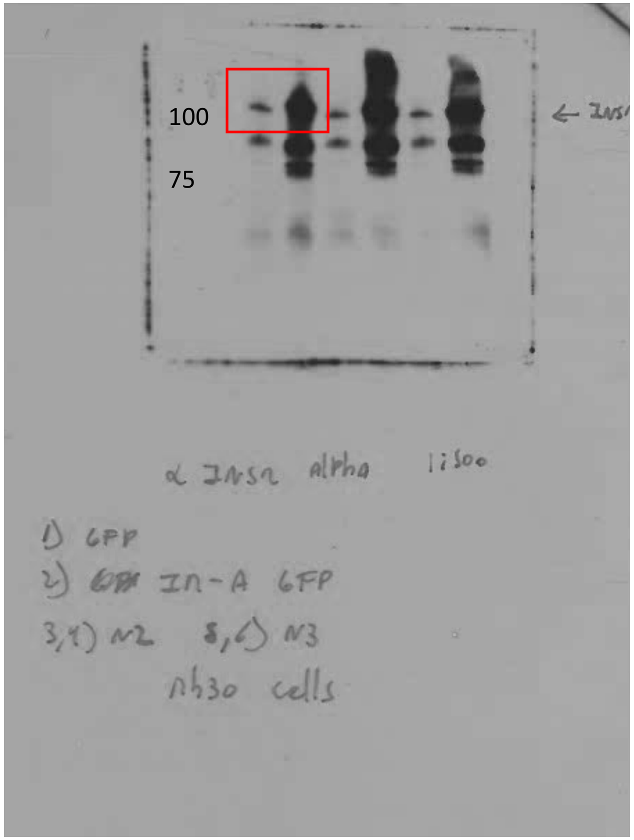

Insulin receptor

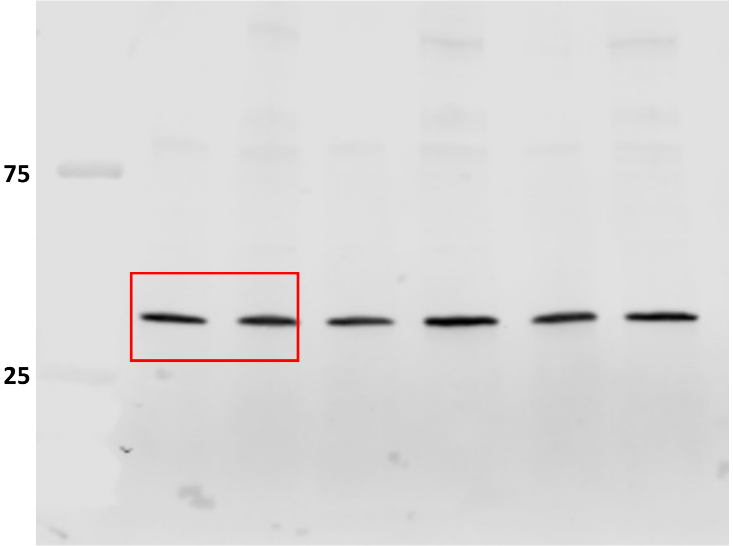

Gapdh

Supplementary Figure 7A

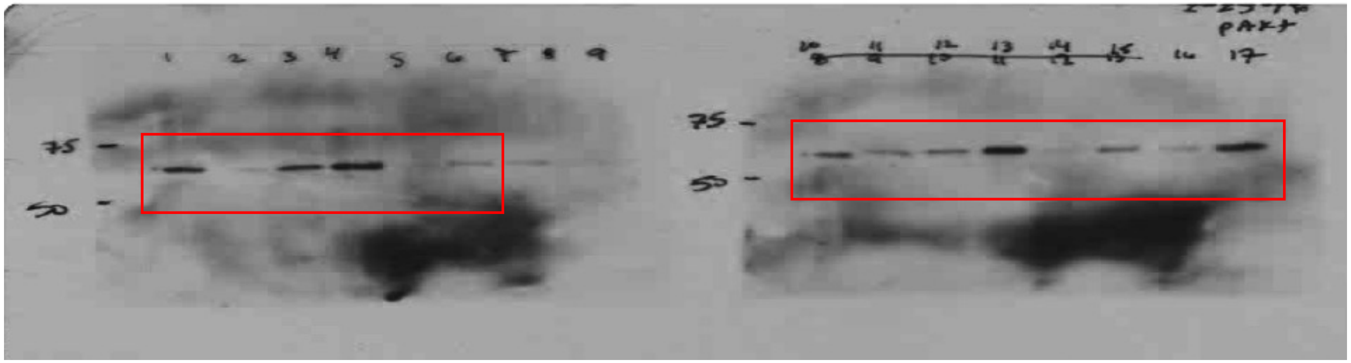

pAKT

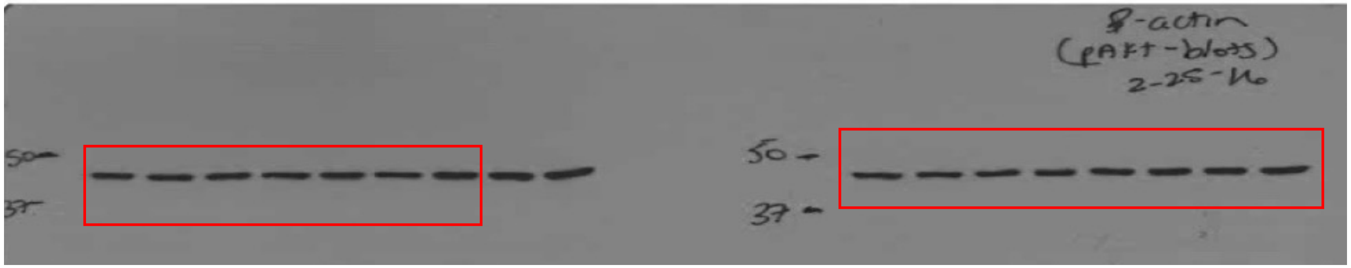

Actin

Supplementary Figure 7A

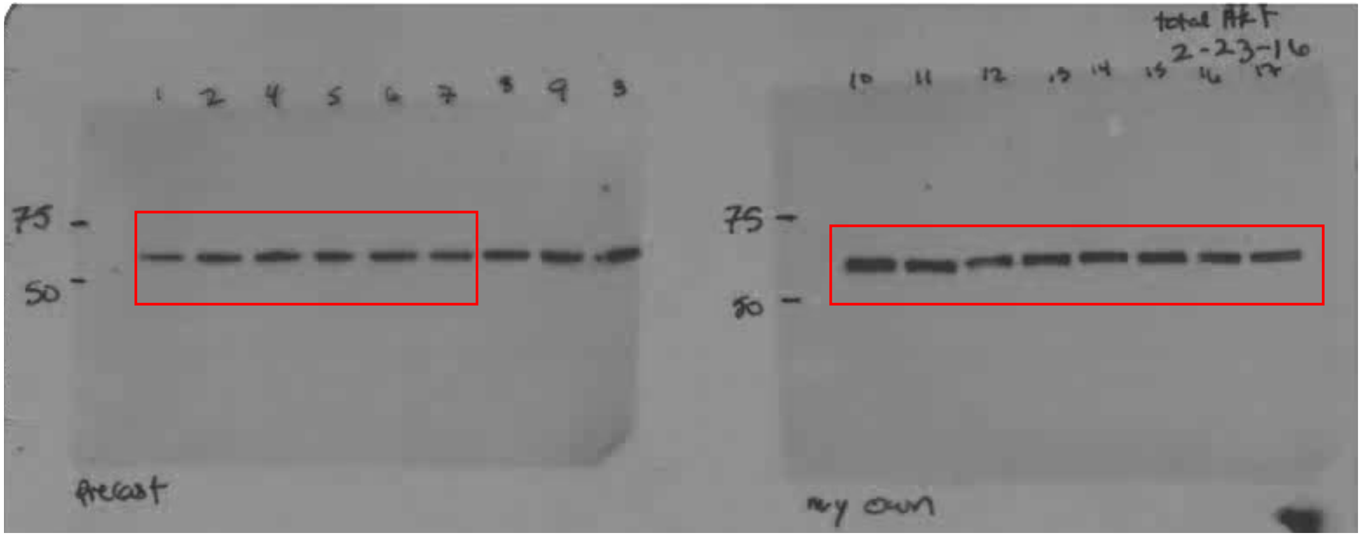

AKT

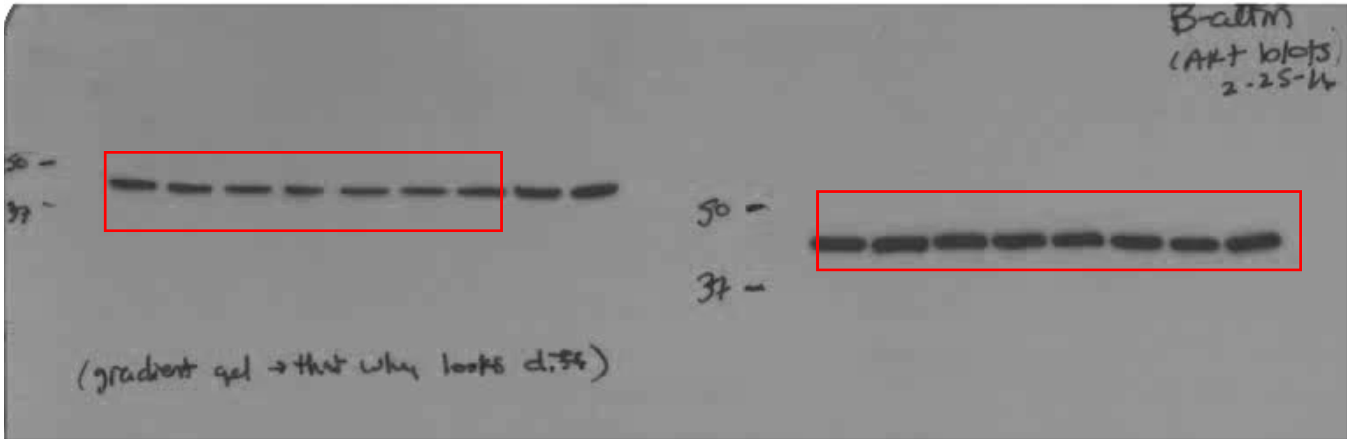

Actin

Supplementary Figure 7B

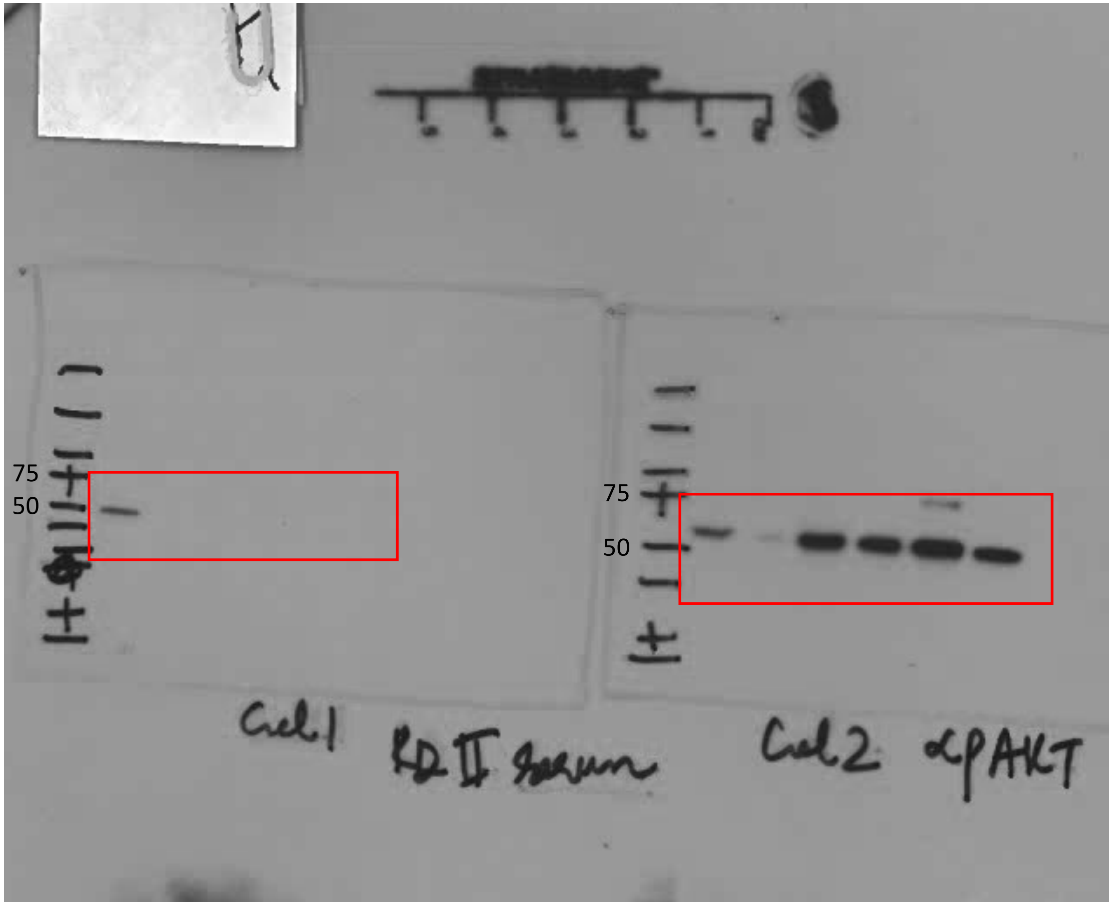

RD serum starvation pAKT

Supplementary Figure 7B

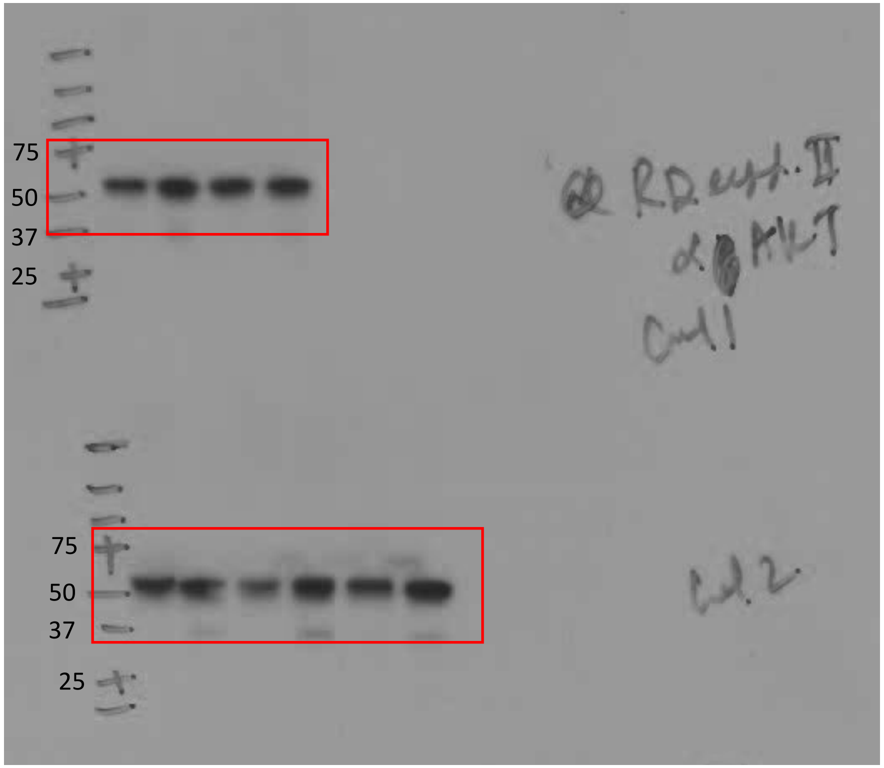

RD serum starvation AKT

Supplementary Figure 7B

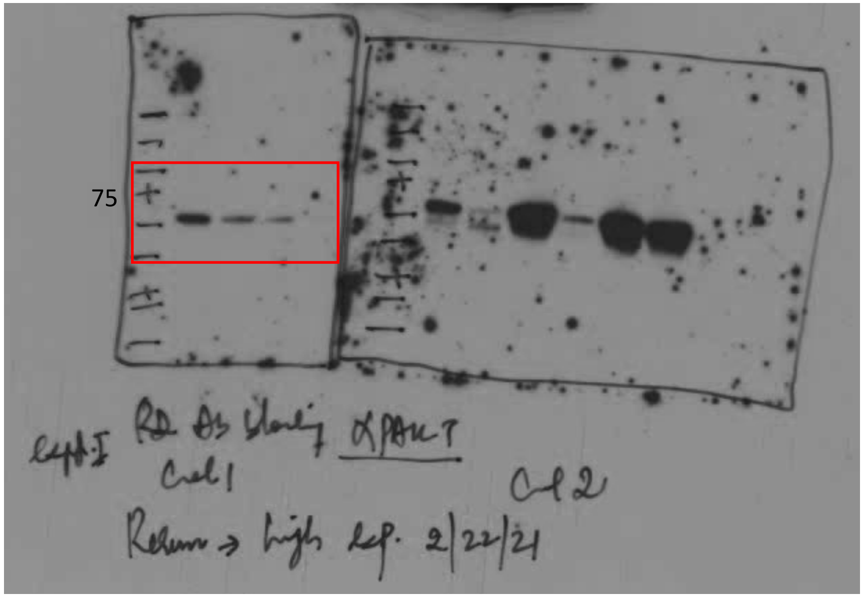

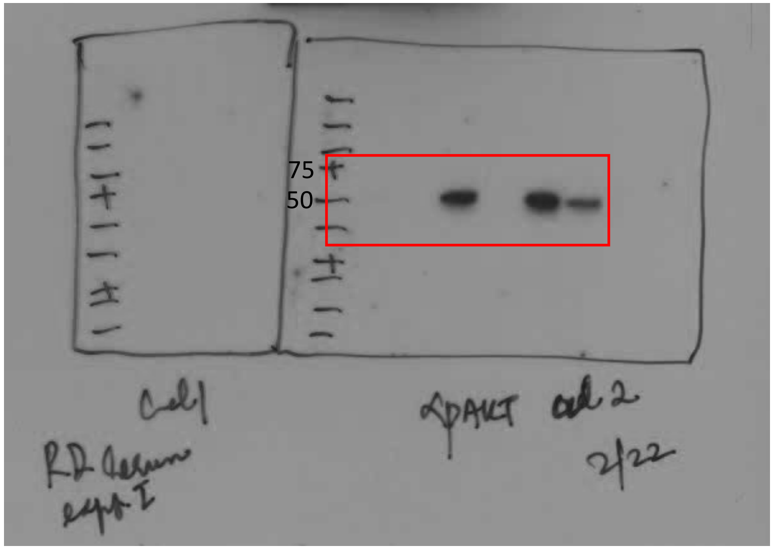

pAKT

Supplementary Figure 7B

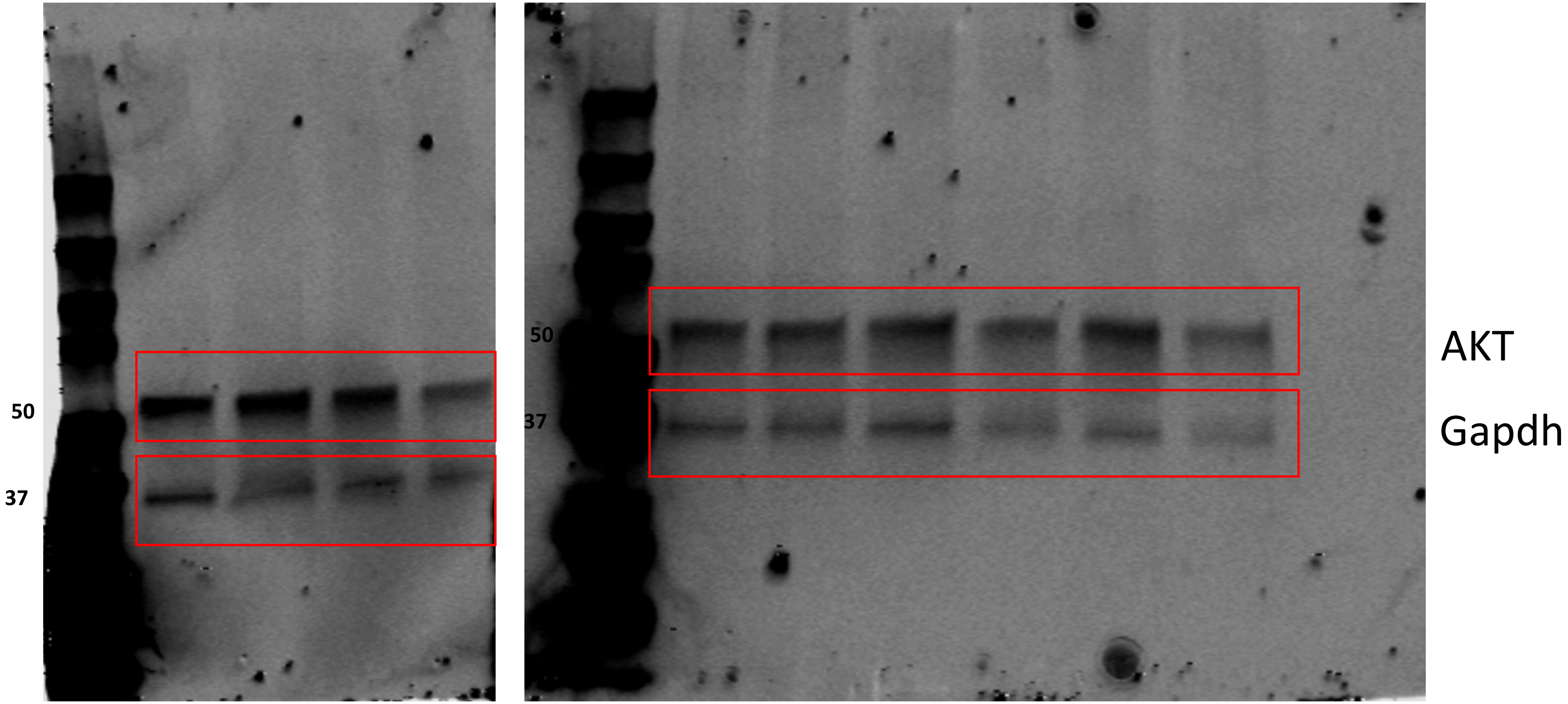

Supplement: Supplementary file 1 — Supplementary figures and tables [file 41698_2021_245_MOESM1_ESM.pdf]
