# Supplementary material for: Splice-switching of the insulin receptor pre-mRNA alleviates tumorigenic hallmarks in rhabdomyosarcoma
Source: NPJ Precis Oncol. 2022 Jan 11;6:1. doi: 10.1038/s41698-021-00245-5 (PMC8752779; doi:10.1038/s41698-021-00245-5)
Supplement: Supplementary file 2 — REPORTING SUMMARY [file 41698_2021_245_MOESM2_ESM.pdf]

nature portfolio

Corresponding author(s): NPJPRECISIONONCOLOGY-01229R

Last updated by author(s): Oct 15, 2021

Reporting Summary

Nature Portfolio wishes to improve the reproducibility of the work that we publish. This form provides structure for consistency and transparency in reporting. For further information on Nature Portfolio policies, see our Editorial Policies and the Editorial Policy Checklist.

Statistics

For all statistical analyses, confirm that the following items are present in the figure legend, table legend, main text, or Methods section.

n/a	Confirmed	
☐	☒	The exact sample size (n) for each experimental group/condition, given as a discrete number and unit of measurement
☐	☒	A statement on whether measurements were taken from distinct samples or whether the same sample was measured repeatedly
☐	☒	The statistical test(s) used AND whether they are one- or two-sided<br>*Only common tests should be described solely by name; describe more complex techniques in the Methods section.*
☐	☒	A description of all covariates tested
☒	☐	A description of any assumptions or corrections, such as tests of normality and adjustment for multiple comparisons
☒	☐	A full description of the statistical parameters including central tendency (e.g. means) or other basic estimates (e.g. regression coefficient) AND variation (e.g. standard deviation) or associated estimates of uncertainty (e.g. confidence intervals)
☒	☐	For null hypothesis testing, the test statistic (e.g. F, t, r) with confidence intervals, effect sizes, degrees of freedom and P value noted<br>*Give P values as exact values whenever suitable.*
☒	☐	For Bayesian analysis, information on the choice of priors and Markov chain Monte Carlo settings
☒	☐	For hierarchical and complex designs, identification of the appropriate level for tests and full reporting of outcomes
☐	☒	Estimates of effect sizes (e.g. Cohen's d, Pearson's r), indicating how they were calculated

Our web collection on statistics for biologists contains articles on many of the points above.

Software and code

Policy information about availability of computer code

Data collection	N/A
Data analysis	Bowtie publically available

For manuscripts utilizing custom algorithms or software that are central to the research but not yet described in published literature, software must be made available to editors and reviewers. We strongly encourage code deposition in a community repository (e.g. GitHub). See the Nature Portfolio guidelines for submitting code & software for further information.

Data

Policy information about availability of data

All manuscripts must include a data availability statement. This statement should provide the following information, where applicable:

- Accession codes, unique identifiers, or web links for publicly available datasets
- A description of any restrictions on data availability
- For clinical datasets or third party data, please ensure that the statement adheres to our policy

All data generated or analyzed are included in the published article (and its supplementary information files).

Field-specific reporting

Life sciences study design

All studies must disclose on these points even when the disclosure is negative.

Sample size	Sample sizes were determined by statistical analyses as described in PPTC papers (for example: Houghton PJ, Lock R, Carol H, Morton CL, Phelps D, Gorlick R, et al. Initial testing of the hypoxia-activated prodrug PR-104 by the pediatric preclinical testing program. Pediatr Blood Cancer. 2010. Epub 2010/12/29.)
Data exclusions	Data exlusions were not utilized for analysis of these data sets
Replication	All replications were successful and included in the manuscript.
Randomization	N/A as all animals used were of the same sex and genotype
Blinding	Blinding was performed for analysis of animal experiments.

Reporting for specific materials, systems and methods

We require information from authors about some types of materials, experimental systems and methods used in many studies. Here, indicate whether each material, system or method listed is relevant to your study. If you are not sure if a list item applies to your research, read the appropriate section before selecting a response.

Materials & experimental systems

n/a	Involved in the study	
☐	☒	Antibodies
☐	☒	Eukaryotic cell lines
☒	☐	Palaeontology and archaeology
☐	☒	Animals and other organisms
☒	☐	Human research participants
☒	☐	Clinical data
☒	☐	Dual use research of concern

Methods

n/a	Involved in the study	
☒	☐	ChIP-seq
☒	☐	Flow cytometry
☒	☐	MRI-based neuroimaging

Antibodies

Antibodies used	MMP-9 (R&D Systems AF911), p-AKT (Cell Signaling 4060), pan AKT (Cell 479 Signaling 4691), GAPDH (Cell Signaling 2118), INSR (Cell Signaling 74118), Hif-1α (BD Biosciences Catalog number 610959), β-Actin clone AC-15 (sigma Aldritch, Catalog 481 Number A5441)
Validation	Antibodies were validated by each manufacturer, data is available in the following websites were additional references are provided: https://www.rndsystems.com/products/human-mmp-9-antibody_af911, https://www.cellsignal.com/products/primary-antibodies/phospho-akt-ser473-d9e-xp-rabbit-mab/4060, https://www.cellsignal.com/products/primary-antibodies/akt-pan-c67e7-rabbit-mab/4691, https://www.cellsignal.com/products/primary-antibodies/gapdh-14c10-rabbit-mab/2118, https://www.cellsignal.com/products/primary-antibodies/insulin-receptor-a-d3u7i-rabbit-mab/74118, https://www.bdbiosciences.com/en-us/products/reagents/microscopy-imaging-reagents/immunofluorescence-reagents/purified-mouse-anti-human-hif-1.610958, https://www.sigmaaldrich.com/US/en/product/sigma/a5441

Eukaryotic cell lines

Policy information about cell lines

Cell line source(s)	RMS cell lines (Rh30, RD, SMS-CTR) cells were obtained from PPTC (see text reference 59). HeLa and HeLa S3 cells were obtained from ATCC.
Authentication	All cell lines derived from human material have been verified by STR analysis by the Labcorp DNA Identification Lab (Burlington, NC USA)
Mycoplasma contamination	All cell lines were tested for mycoplasma contamination using the universal mycoplasma detection kit sold by ATCC (Catalog number 30-1012K)
Commonly misidentified lines (See ICLAC register)	*Name any commonly misidentified cell lines used in the study and provide a rationale for their use.*

Animals and other organisms

Policy information about studies involving animals; ARRIVE guidelines recommended for reporting animal research

Laboratory animals	Envigo CB17 SC-/- SCID mice were used for matrigel grafts. (https://www.envigo.com/model/c.b-17-icrhsd-prkdcscid)
Wild animals	N/A
Field-collected samples	N/A
Ethics oversight	N/A

Note that full information on the approval of the study protocol must also be provided in the manuscript.
